# Supplementary material for: Perspectives of informal caregivers who support people following hip fracture surgery: a qualitative study embedded within the HIP HELPER feasibility trial
Source: BMJ Open. 2023 Nov 17;13(11):e074095. doi: 10.1136/bmjopen-2023-074095 (PMC10660837; doi:10.1136/bmjopen-2023-074095)
Supplement: Supplementary data [file bmjopen-2023-074095supp001.pdf]

**Topic**	**Sample questions**	**Prompts/probes**
Overall	• Overall, do share your experiences of being involved with our research?	
The approach and consent process	• Please talk me through how you got involved with the HipHelper research study?<br>• How clearly was the study explained to you?	• Could we have dealt with that differently?<br>• How was this different to what you expected?<br>• For the future, what could we improve about the description of the study?
Willingness to be randomised to either group/ participant views of their intervention<br><br>The risk of intervention contamination between the groups	• Please talk me through what study treatment you received<br><br>• How much did talk to any other patients or caregivers (whilst in hospital) about the intervention?	• *If required: clarify what HIP HELPER was and what was usual care/non-study intervention*<br>• You were allocated to X group. What did that feel like?<br>• What discussions were had between those who received it and did not receive it?
**Both groups**		
The acceptability of the inpatient care	• Please talk me through your treatment while you were in the hospital	• What aspects of your care that were lacking in the hospital?<br>• Can you remember which wards you were on? Can you remember being moved to different areas of the hospital?

**Topic**	**Sample questions**	**Prompts/probes**
	• *(Carers)* As X's carer, what was your impression of the care?	• For both of you, what was helpful and less helpful to your care?
**Intervention group only**		
In-patient HIP HELPER programme<br><br>Strengths/weaknesses	• How did you find the HIP HELPER programme?<br><br><br><br><br><br><br>• Please tell me what you remember about the manual handling training you had in the hospital?<br>• Please tell me about the phone calls you received?<br><br><br><br>• What were the individualised goals you set in the hospital?	• Please share any specific examples of what you can remember from the programme?<br>• What were the most helpful bits of your HIP HELPER intervention?<br>• What was good about it?<br>• What were the less helpful/worse bits of the HIP HELPER intervention?<br>• For you as the patient/and you as X's carer?<br>• How did you get on with that at home?<br>• Was it helpful? If so, how?<br><br>• Can you remember what you talked about?<br>• Can you give specific examples of what was helpful?<br>• Was there any advice that confused you or you weren't clear about?<br>• How did you come up with these?<br>• *(Carer)* By how far were you involved in the goal setting process?<br>• In retrospect, what goal(s) would you alter?

Topic	Sample questions	Prompts/probes
**Both groups:**		
On return to your home	• *(If required, state residential status)* Can you tell me who you live with at home?	
	• What were the first things you wanted to achieve once at home?<br>• How did you decide who was to be your allocated carer?<br>• *(Carers)* Can you talk me through this decision to be designated as X's carer?	• On a 1-10 scale, how confident did you feel when you left the hospital?<br>• Can you tell me why you have chosen that number on the scale?
	• *(Long-term carers)* Once at home, how do you feel care-giving role changed?	• Can you tell me about any new caring responsibilities?<br>• How did you organise these?<br>• Did this change the dynamics in their relationship in taking on caring role, if so, how
	• What sort of adaptions did you make at home to enable you to move about independently (where possible)?<br>• What do you think is lacking in the transition from hospital to home?	• Are/were there any other things that may have affected your transfer home?<br>• Are/were there any other things that required you to adapt the advice you were given?
	• How willing were/are you to continue with activities/advice that were suggested to you?<br>• Once at home, by how far did you feel supported by the health care professions you had seen?	*Probes: transferring, dressing, activities of daily living*

Topic	Sample questions	Prompts/probes
	• *(Carers)* For you as caregiver, how did you get on with helping move X about?<br>• Were there any adaptions that you made to the advice given?	
What modifications they may recommend to interventions received	• What changes did you find easy to implement and what did you find harder?	• What could we improve?
	• What do we need to modify for any future programmes to support people with a hip replacement and their carer to help you get up and moving and returning to normal life?	• Can you explain why? *Prompt around ADL's*
The ease and convenience of the data collection processes/applicability of the methods and measures used	• As you were part of a trial, we had to collect a lot of measurements. Can you talk me through what these were?	*Prompt:*<br>*'Share screen' of the front page of questionnaires to help with memory recall.*
	• How did you manage with the questionnaires we gave you at the start of the study and at the end in the post?<br>• What about your interview experience today?	• Were they easy to complete?<br>• Do you remember them being a problem?<br>• How convenient were they?<br>• How did you find it?<br>• Is there anything we can do to improve this experience?
Summary and end	• We are now ? months since your hip replacement, by how far do you think the HipHelper programme (*or usual care)* has helped with your recovery?<br>• If we were to run this programme again in the future, is there anything else we should consider?	• How far do you think the Hip Helper programme (*or usual care)* has helped you achieve your goals?

Topic	Sample questions	Prompts/probes
	• How do you think we could better support you and your carer to recover after hip surgery?<br>• Is anything else you have gained by being involved in this research study<br>• Is there anything else we haven't thought of and that you can share with us to improve the study and the care of hip replacement patients and their carers?	
