# Supplementary material for: Perspectives of informal caregivers who support people following hip fracture surgery: a qualitative study embedded within the HIP HELPER feasibility trial
Source: BMJ Open. 2023 Nov 17;13(11):e074095. doi: 10.1136/bmjopen-2023-074095 (PMC10660837; doi:10.1136/bmjopen-2023-074095)
Supplement: Author's manuscript [file bmjopen-2023-074095.draft_revisions.pdf]

BMJ Open is committed to open peer review. As part of this commitment we make the peer review history of every article we publish publicly available.

When an article is published we post the peer reviewers' comments and the authors' responses online. We also post the versions of the paper that were used during peer review. These are the versions that the peer review comments apply to.

The versions of the paper that follow are the versions that were submitted during the peer review process. They are not the versions of record or the final published versions. They should not be cited or distributed as the published version of this manuscript.

BMJ Open is an open access journal and the full, final, typeset and author-corrected version of record of the manuscript is available on our site with no access controls, subscription charges or pay-per-view fees (http://bmjopen.bmj.com).

If you have any questions on BMJ Open's open peer review process please email info.bmjopen@bmj.com

BMJ Open

Caregiver perspectives of supporting people following hip fracture surgery: qualitative study from the HIP HELPER trial.

Journal:	*BMJ Open*
Manuscript ID	bmjopen-2023-074095
Article Type:	Original research
Date Submitted by the Author:	27-Mar-2023
Complete List of Authors:	Welsh, Allie; University of East Anglia, School of Education<br>Hanson, Sarah; University of East Anglia, School of Health Sciences<br>Pfeiffer, K; Robert Bosch Hospital, Department of Clinical Gerontology and Geriatric Rehabilitation<br>Khoury, Reema; University of East Anglia,<br>Clark, Allan; University of East Anglia, Norwich Medical School<br>Ashford, Polly-Anna; University of East Anglia, Norwich Clinical Trials Unit<br>Hopewell, Sally; University of Oxford, Nuffield Department of Orthopaedics, Rheumatology and Musculoskeletal Sciences<br>Logan, Phillipa; University of Nottingham, Community Health Sciences<br>Crotty, Maria; Flinders University, Rehabilitation, Aged and Extended Care<br>Costa, Matthew; University of Oxford, Nuffield Department of Orthopaedics, Rheumatology, and Musculoskeletal Sciences<br>Lamb, Sarah; University of Exeter, College of Medicine and Health<br>Smith, Toby; University of Warwick; University of East Anglia, School of Health Sciences
Keywords:	Hip < ORTHOPAEDIC & TRAUMA SURGERY, QUALITATIVE RESEARCH, REHABILITATION MEDICINE

SCHOLARONE™
Manuscripts

I, the Submitting Author has the right to grant and does grant on behalf of all authors of the Work (as defined in the below author licence), an exclusive licence and/or a non-exclusive licence for contributions from authors who are: i) UK Crown employees; ii) where BMJ has agreed a CC-BY licence shall apply, and/or iii) in accordance with the terms applicable for US Federal Government officers or employees acting as part of their official duties; on a worldwide, perpetual, irrevocable, royalty-free basis to BMJ Publishing Group Ltd ("BMJ") its licensees and where the relevant Journal is co-owned by BMJ to the co-owners of the Journal, to publish the Work in this journal and any other BMJ products and to exploit all rights, as set out in our licence.

The Submitting Author accepts and understands that any supply made under these terms is made by BMJ to the Submitting Author unless you are acting as an employee on behalf of your employer or a postgraduate student of an affiliated institution which is paying any applicable article publishing charge ("APC") for Open Access articles. Where the Submitting Author wishes to make the Work available on an Open Access basis (and intends to pay the relevant APC), the terms of reuse of such Open Access shall be governed by a Creative Commons licence – details of these licences and which Creative Commons licence will apply to this Work are set out in our licence referred to above.

Other than as permitted in any relevant BMJ Author's Self Archiving Policies, I confirm this Work has not been accepted for publication elsewhere, is not being considered for publication elsewhere and does not duplicate material already published. I confirm all authors consent to publication of this Work and authorise the granting of this licence.

For peer review only

BMJ OPEN

TITLE PAGE

Title: Caregiver perspectives of supporting people following hip fracture surgery: qualitative study from the HIP HELPER trial.

Authors: Welsh A,[1] Hanson S,[2] Pfeiffer K,[3] Khoury R,[2] Clark A,[2] Ashford P-A,[2] Hopewell S,[4] Logan PA,[5] Crotty M,[6] Costa ML,[4] Lamb SE,[7] Smith TO[2,8] on behalf of the HIP HELPER Study Collaborators.

Affiliations

1. School of Education, University of East Anglia, Norwich, UK
2. Faculty of Medicine and Health Sciences, University of East Anglia, Norwich, UK
3. Department of Geriatric Rehabilitation, Robert-Bosch-Hospital, Stuttgart, Germany
4. Nuffield Department of Rheumatology, Orthopaedics and Musculoskeletal Sciences, University of Oxford, Oxford, UK
5. School of Medicine, University of Nottingham, Nottingham, UK
6. College of Medicine and Public Health, Flinders University, Adelaide, Australia
7. College of Medicine and Health Sciences, University of Exeter, Exeter, UK
8. Warwick Medical School, University of Warwick, Coventry, UK

Corresponding Author: Dr Allie Welsh, School of Education, University of East Anglia, Norwich, NR4 7TJ. Email: a.welsh@uea.ac.uk

For peer review only

ABSTRACT

Objectives: To illuminate the perspectives of informal caregivers who support people following hip fracture surgery.

Design: A qualitative study embedded within a multi-centre, feasibility randomised controlled trial (HIPHELPER).

Setting: Five English National Health Service (NHS) hospitals.

Participants: We interviewed 20 participants (10 informal caregivers; 10 people with hip fracture), following hip fracture surgery. This included one male and nine females who experienced a hip fracture; and seven male and three female informal caregivers. The mean age was 72.5 years (range: 65 to 96 years), 71.0 years (range: 43 to 81 years) for people with hip fracture and informal caregivers, respectively.

Methods: Semi-structured, virtual interviews were undertaken between November 2021 and March 2022, with caregiver dyads (person with hip fracture and their informal caregiver). Data were analysed thematically.

Findings: We identified two main themes: expectations of the informal caregiver role, and reality of being an informal caregiver; and sub-themes: expectations of care and services; responsibility and advocacy; profile of people with hip fracture; decision to be a caregiver; transition from hospital to home.

Conclusion: Findings suggest informal caregivers do not feel empowered to advocate for a person's recovery or navigate the care system, leading to increased and unnecessary stress, anxiety and frustration when supporting the person with hip fracture. We suggest that a tailored information-giving on the recovery pathway, which is responsive to the caregiving population (i.e., considering the needs of male, younger, and more active informal caregivers and people with hip fracture) would smooth the transition from hospital to home.

Key words: informal caregivers; hip fracture; recovery; rehabilitation; carer; qualitative

Trial registration number: ISRCTN13270387

Data availability statement: The data that support the findings of this study are available from the corresponding author (SH) upon reasonable request. This includes access to the full protocol, and anonymised participant-level dataset.

For peer review only

STRENGTHS AND LIMITATIONS OF THIS STUDY

- We describe the contextual factors of participating in HIP HELPER and people's experiences of giving and receiving informal care.
- This study may help health professionals support informal caregiver dyads in the transition from hospital to home.
- Participants presented with limited socioeconomic, cultural or ethic perspectives, and there was a lack of perspectives of those caring for a person with cognitive impairment.
- COVID-19 pandemic affected NHS services which may have impacted on the study delivery and participant's experiences.

KEY HIGHLIGHTS/IMPLICATIONS

- There is a tension between the expectations, and realities of the caregiving role for people with hip fracture.
- Empowering informal caregivers with information, joint decision-making and goal-setting will enable them to better advocate for people with hip fracture.
- Giving tailored information on the recovery pathway will likely improve the transition to home.
- Greater consideration could be given to younger more active hip fracture patients who have different expectations of recovery goals.

Word Count: Manuscript: 3735; Abstract: 250

For peer review only

INTRODUCTION

Hip fracture is a devastating injury, predominantly seen in frail older people, and disproportionally more women [1]. Approximately 80,000 people aged 60 years and over experience a hip fracture in the UK each year, resulting in a combined health and social cost of at least £2 billion [2]. This cost is expected to increase with an ageing population [1]. Outcomes following hip fracture are poor. The majority of individuals do not return to pre-injury levels of function, and frequently lose independence and self-caring [3,4].

Given the sudden, traumatic nature of hip fracture, people often rely on family members or friends for informal care following hospital discharge. Additionally, approximately 40% of people who sustain a hip fracture have cognitive impairment [5], meaning many caregivers also have to cope with the challenges of functional dependency and neurocognitive symptoms. These 'informal caregivers' therefore play an integral role, in the transition from hospital to home, the initial discharge period and possibly beyond [6].

Informal caregivers are a heterogeneous population. For some, this may be the first time with caregiving responsibilities; for others, it is perceived as an episodic event with a temporary increase in care needs. They may be a retired spouse, friend, grown-up children in employment; living with, close by or far away; and of course, they be facing their own physical and mental health challenges. Caregiving may further be exacerbated by (but not limited to) the ageing population, smaller average family size, changing gender-roles due in part to more women working outside the home [7–9].

The ability of a person to cope with the roles and responsibility of caregiving is a complex interaction of different factors. In addition to stressors related to the actual caregiving, there are other factors that may determine a person's ability to manage the caregiving role, such as: caregiver resources, social environment (e.g., informal and professional support), relationship quality with the care-recipient and caregiver, positive aspects of caregiving (e.g., finding meaning in the caregiving role) [10]. Whilst caregiving experiences have been reported in other populations such as stroke [6] and dementia [11], there is still much to understand about acute episodes of informal care, such as in hip fracture.

Our qualitative study investigated the informal caregiver perspectives of caring for a person with hip fracture following hospital discharge, nested within a larger study aimed at assessing the feasibility of a pragmatic, multi-centre randomised controlled trial (RCT) of an informal caregiver training programme (HIP HELPER) to support the recovery of people following hip fracture surgery [12].

METHODS

This was a qualitative study embedded within a multi-centre, feasibility randomised controlled trial (HIP HELPER). Detailed methods of the HIP HELPER study are published elsewhere [12]. In brief, HIP HELPER aimed to assess the design of a pragmatic RCT to test the effectiveness of an informal caregiver intervention, compared to usual NHS care following hip fracture surgery. Caregiver dyads who were randomised to receive the HIP HELPER intervention were allocated to received three, one- hour, one-to-one training sessions, delivered by a nurse, physiotherapist, or occupational therapist. Training sessions included practical skills for rehabilitation such as: transfers and walking, pacing, and stress management techniques, and the provision of and the HIP HELPER Caregiver Workbook, offered information on recovery, exercises, worksheets, and goal-setting plans to facilitate a 'good' recovery.

For peer review only

The qualitative sub-study aimed to explore the experiences and contextual factors of participating in HIP HELPER and their experiences of giving and receiving informal care [13] and followed the COREQ reporting guidelines [14].

Caregiver dyads were purposively sampled by age, pre-fracture disability, and hospital locations. An informal caregiver was defined as someone who was expected to informally provide care, assistance, support or supervision in activities of daily living (ADLs) for at least three hours per week, but not on a paid basis. This may have included personal ADLs such as toileting, washing, dressing and eating; and/or more complex tasks such as managing money, shopping and household chores [15,16].

Data Collection

In-depth, semi-structured interviews were undertaken with caregiver dyads, within six-weeks of hospital discharge, by AW, who is a white, female post-doctoral researcher. AW had no role in recruitment to the study nor intervention delivery, thus was not known to participants. Interviews were conducted virtually using Microsoft Teams or via telephone, between November 2021 and March 2022. They were audio-recorded, transcribed and all identifying information removed. The topic guide was developed with two members of the public, who helped guide the approach and style of the interview topic guides through a pilot interview (**Supplementary File 1**).

Data Analysis

Data were initially analysed deductively against our questioning framework and then further explored inductively to explore contextual features and participants experiences [13]. We used the principles of thematic analysis with regular discussion among the team (AW, SH, TS, KP) to develop our key themes [17].

FINDINGS

Participants

In total, 10 interviews were undertaken with caregiver dyads (20 participants) across our five study sites in England. Characteristics are presented in **Table 1**. The mean age of people with hip fracture and their caregiver was 72.5 years (range: 65 to 96 years) and 71.0 years (range: 43 to 81 years), respectively. Of the informal caregivers, seven were male and three were female. There were three males and seven females who experienced a hip fracture. Overall, seven informal caregivers were the spouse of the person with hip fracture, two were adult children (a daughter and a son) and two were described as 'other'.

Themes

Overall, two main themes and associated sub-themes were identified:

(1) Expectations of the informal caregiver role
   a) Expectations of care and services
   b) Responsibility and advocacy

(2) Reality of being an informal caregiver
   a) Profile of people with hip fracture
   b) Decision to be a caregiver
   c) Transition from hospital to home

For peer review only

Expectations of the informal caregiver role

Informal caregivers perceived that health professionals assumed that they would take-on (additional) caregiving responsibilities upon the discharge home. Such findings are comparable to literature exploring caregiver perceptions of caring for a person with stroke, as there is an immediate demand to assume a role of significant responsibility [18].

"Assumed that because I'm working from home [I would care], just said it like, didn't even ask me." (Caregiver, Female)

a) Expectations of care and services

There was a feeling that rather than being personalised, goals were set by health professionals on a 'standard' patient recovery trajectory and therefore these were not viewed as achievable, nor realistic. They also had insufficient information to understand what a typical recovery trajectory looks like. It may therefore be understandable that people did not feel empowered and found goal-setting and developing realistic recovery goals difficult, based on imperfect information.

"I had no expectations because I have never been in hospital before. Ever. I have never been ill before, so this is completely new to me and obviously new to [caregiver] – he's never had to care for me before." (Person with Hip Fracture, Female)

"Not quite enough available to you about kind of next steps and what's going to happen in the future." (Caregiver, Male)

In contrast, receiving knowledge on recovery expectations with realistic goal-setting and joint decision-making, as seen in our HIP HELPER intervention, appeared to be empowering.

"If you get somebody who doesn't have sufficiently stimulating support, that is a problem because in a sense, it encourages them to stay dependent, whereas what it should be doing is encourage them to become independent." (Caregiver, Male)

b) Responsibility and advocacy

Caregivers reported a sense that they must advocate and even battle for services to achieve adequate care and support in the transition from hospital to home, and yet, they had no power to do so. Navigating the care system is difficult and requires knowledge and confidence. This was particularly pertinent among first-time caregivers.

"There is only as much as I can do to protect my husband. He's got a right to have a minimum care. Really, we're not asking for much." (Caregiver, Female)

"I had to make phone calls and chase around to try and sort things out [for her recovery at home]." (Caregiver, Female)

This level of advocacy was time consuming and created an imbalance in undertaking care activities, thus detracting from giving personal support. This is a common theme that resonates within previous literature [15,19,20].

For peer review only

"But while I have to chase up all these things, he's not getting the best. He needs my attention, you know, to make sure that he's doing his exercise." (Carer, Female)

Caregivers also expressed their feelings of accountability for supporting independence and recovery and, not surprisingly for some, this induced feelings of stress and anxiety.

"She's always been very independent, and I just felt very responsible for it. Seems to be quite a lot on my shoulders." (Caregiver, Male)

"There was a shock when she fell and broke her hip. It's a lot to take on. All of a sudden, everything falls to one person, it's tough." (Caregiver, Male)

Reality of being an informal caregiver

There was a dissonance in the expectations of being a caregiver and the actual role. The reality was shaped by the motivation and willingness to become a caregiver, the profile of the caregiver and the person with hip fracture, and the process of being discharged home. This mismatch was seen more in first-time caregivers and spouses of the person with hip fracture.

a) Profile of the caregiver and the person with hip fracture

The majority (n=8/10) of caregivers were first-time caregivers with little or no understanding of the hip fracture recovery process. This is contrary to experienced caregiver who could elicit competence, knowledge, and tolerance in caregiving [21–24]. Additionally, the profile of a caregiver may include a natural affinity to caring, namely a 'caring nature' [25].

"His wife had Alzheimer's and he cared for her for about 16 years. So, it's something that he understands. And he, well, he's just very good at it." (Person with Hip Fracture, Female)

For those caregivers in full-time employment, competing priorities between work and caring highlighted the reality of being an informal caregiver. This is an important area for discussion as previous literature suggests that caregivers who are in employment may experience constrained motivations to care, resulting in a source of tension [26].

"I had loads of work on and I was having to work at 10 o'clock at night to try and get my work done, because through the day when places were open, I was fooling around trying to get the things we need, sort mam and do her appointments." (Caregiver, Female)

The profile of people with hip fracture in this study, were generally active older adults, some of whom were still in employment and felt the services available post-hip fracture, were not conducive to their lifestyle, needs or goals. They perceived services are primarily designed for older adults, suggesting inadequacy in rehabilitation for those with hip fracture (and their caregivers) aiming to return to occupational or physically-demanding leisure activities.

*"I don't think we're typical patients and carers. Certainly, I am definitely not a typical carer, and *** is definitely not a typical patient."* (Caregiver, Male)

"You know, I'm not a little old lady who sits and knits in the chair and doesn't move about much so that makes a difference, doesn't it?" (Person with Hip Fracture, Female)

For peer review only

Griffiths et al [27] reported similar findings in that there was considerable variation of what was considered important in recovery within a broad hip fracture population. For example, younger, otherwise healthy people who experience hip fracture, emphasised returning to their pre-fracture functional state, as opposed to different rehabilitation goals for a person who is frail, or nearing the end of life.

Caregivers were also mindful of their own age and health status. Caregivers were aware that they required physical and mental capacity to effectively care for the person following hip fracture and that maintaining their own health and wellbeing was equally as important.

*"I was conscious that I had to take care of myself to be fit to be looking after her." (*Caregiver, Male)

"You know I'm so lucky that I'm healthy still. That I'm young and capable, but, who knows, you know?" (Caregiver, Male)

Much like Zarzycki et al (26), caregivers recognised the need to balance their own physical and psychological needs with their care-recipient's need [26]. There is a growing body of literature suggesting the mental health and wellness of a caregiver is also linked with a patient's functional and health outcomes [28–30]. Maintaining physical and mental ability to care for an individual is therefore important for hip fracture recovery.

b) Decision to be a caregiver

Our findings are consistent with Zarzycki's systematic review of 103 qualitive studies synthesising personal and relational motivations for providing informal care [26]. They identified multiple factors which contributed to the decision to become an informal caregiver. Spousal obligation and acting out of affection, were examples identified from the present study. For some, values such as loyalty and commitment arising from marriage (spousal obligation) was what motivated people to take on a caregiving role [26].

"We both met two and a half years ago. Dare I say it nuts about each other? Ever since then, we've been living together, as man and wife." (Caregiver, Male)

For others, there was the absence of *actual choice*, in-part attributed to assumptions made on discharge planning, but also underpinned by the unavailability of alternative care options.

"I think in our case there wouldn't have been anyone else who would be able to come and help." (Caregiver, Male)

An interesting point about the decision to be a caregiver is that no participant expressed an awareness of caring for a limited acute period of time. Thus, it is unknown how long caregivers perceive the responsibility of caring for a person with hip fracture lasts for.

c) Transition from hospital to home

Caregivers expressed frustration, confusion, and uncertainty at the point of transitioning home. Such feelings were principally attributed to a lack of communication throughout discharge planning with caregivers feeling isolated, under-prepared and excluded from the decision-making. Visitor restrictions due to the COVID-19 pandemic, may have also reduced communication channels and

For peer review only

contributed to caregiver uncertainty, resulting in concerns of whether the person with hip fracture was receiving adequate care [31].

> *"It's very difficult. I was at one point sending her back to hospital 'cause I was so frantic with it all."* (Caregiver, Female)

> *"Nothing is said [about discharge] at the hospital, apart from telling you have an appointment in eight weeks, there's nothing else said."* (Caregiver, Male)

> *"I mean the one thing that I do remember is really actually a feeling of isolation and a little bit concerned at whether or not I was doing the right things to support her."* (Caregiver, Male,)

The profile of caregivers also contributes to their competence in managing the transition home, potentially relying on previous experiences to navigate the health system and available services.

> *"You know, imagine if you if you weren't an old nurse like me? How on earth can you even navigate the system? I think people are getting lost out there and it's creating a lot of problems for the health service."* (Caregiver, Female)

Some participants shared how they had to 'beg, steal and borrow' equipment from friends and family. It is apparent that the onus is on the caregiver themselves to navigate and seek the appropriate provisions for recovering at home with hip fracture and there was a feeling of abandonment.

> *"Luckily, some family had a spare single bed. A friend had a perching stool and a trolley. And then another friend, sadly her husband had been ill, but they had purchased their own wheelchair and we actually borrowed that wheelchair. And a cousin, he built some ramps so I could get the wheelchair in."* (Caregiver, Female)

DISCUSSION

A previous study by Tutton et al (31) has provided important insights into the experiences of patients and caregivers following hip fracture, which we have further explored to better understand this challenge. Our study aimed to understand the perspectives of informal caregivers who support the recovery of people following hip fracture surgery. Findings indicate that there is a tension between the expectations, and reality of the caregiving role for people with hip fracture. This is largely attributed to caregivers feeling disempowered to advocate for a person's recovery, leading to increased stress, anxiety and frustration when supporting them. Additionally, caregivers struggle with 'juggling' their own life with their caregiving role, whilst also maintaining their own health and well-being, for the benefit of both members of the dyad when discharged home.

This study identified that discharge processes after hip fracture do not currently fully prepare informal caregivers for their roles. As such, key learning points for health professionals are suggested to improve collaboration and support in the transition from hospital to home. These include: a greater explanation on 'normal' recovery processes; engaging for (dyad) joint decision-making; and promote the provision of additional (community) services for support. This is supported by previous findings that has also highlighted several key recommendations for translational care which emphasise dyad engagement, education and wellbeing [32,33].

Joint decision-making based on setting realistic and tailored goals, is another practical strategy health professionals may employ to support the transition of people with hip fracture and their informal

For peer review only - http://bmjopen.bmj.com/site/about/guidelines.xhtml

caregiver from hospital to home. This is in agreement with previous authors such as Angeli et al (35) and Brewer et al (34), who stressed that collaborative goal-setting can allow designated time to enhance family involvement, to focus on functional outcomes and highlight current priorities for the caregiving dyad. Findings from the present study highlight the position of informal caregivers as 'drivers of goal-setting', as they may have intimate knowledge of the person pre-hip fracture (for example, preferences and character).

A key finding of this study was the powerlessness which caregivers felt they had in supporting and advocating for the person with hip fracture. As a result of their inability to advocate for the person with hip fracture, participants frequently reported feelings of either over anticipation (negative) contrasted with unrealistic (positive) opinions on what could be offered and provided to the person who they were supported. There was an expectation by health professionals that caregivers could navigate the health and social care system, which would indeed, require some level of health literacy. It is known that low health literacy among caregivers has the potential to impact adequate care provision [34]. This is particularly pertinent among those who are 'new' caregivers, those who may perceive to be particularly under-skilled, under-prepared and/or be adjusting to a new role or identify as an informal caregiver. There is therefore the need to develop interventions that improve informal caregiver health literacy, ultimately upskilling informal caregivers to have the confidence to advocating for the person with hip fracture.

This study offers insights into an informal caregiver population which may differ to most previously reported. Previous literature has emphasised the caregiver experience of caring for people with long-term conditions (e.g., MacDonald et al (37)) which is unlikely to be transferable to the acute, traumatic and rapid nature of caregiving for people with hip fracture. This study therefore offers unique perspectives on a different transition in becoming a (informal) caregiver, particularly as the findings suggest little time for decision-making. A second element to this caregiving population is how it is gendered. Caregiving is known to have a greater adverse effect on female caregivers' mental health and life satisfaction [35]. However, the demographic of hip fracture is largely female (considering experiences of the menopause and osteoporosis, to name a few) [36], as seen in our larger study necessitating male partners to take on the caring role who may find undertaking sensitive tasks more challenging [37]. Additionally, the qualities and skills frequently offered by female caregivers previously reported are that females are better communicators, better instigators of help and are better organised, suggesting that males caregivers might benefit with tailored support for these potential challenges [25]. Whilst it is recognised that expertise in managing frail, older patients is crucial to ensure holistic care for hip fracture patients [38], our study also featured much younger hip fracture participants, who had quite different expectations of recovery and rehabilitation needs.

This study presents with strengths and limitations. A notable strength was its ability to capture experiences during a novel time in healthcare history, where hospitals were making rapid, visitor policy changes in response to the COVID-19 pandemic. Due to the timing of the study, the participants shared a distinctive experience and its effect rehabilitation and on the caregiver experience. Interviews took place within four-months post-hip fracture, despite previous literature suggesting physical recovery may take up to 12 months [39], and high levels of caregiver burden reported by caregivers at six months post-surgery [20]. There is currently little data regarding caregiver perspectives on the long-term recovery/care of hip fracture (for example, deconditioning and confidence in performing activities), thus understanding perspectives of the different caregiving trajectory, as people become more experienced carers and as people recover from hip fracture, would be valuable. Furthermore, it is unknown how long caregivers perceive the responsibility of caring for a person with hip fracture lasts for. No participant expressed an awareness of caring for an acute period of time and therefore how long this might last [40]. We acknowledge findings may not be fully generalisable because of the small sample size and England-based focus. Participants presented with

For peer review only

limited socioeconomic, cultural or ethic perspectives, despite our best endeavours and there was also a lack of perspectives of those caring for a person with cognitive impairment [41]. This is important as such differences may be a source of variation in attitudes, behaviours and perspectives of caregiving for someone following hip fracture [42,43]. We also appreciate that the present cohort was largely of lower mean age than may be expected for a hip fracture population, however this work provides more novel insights into the experiences of caregiving for younger people which is a profile anecdotally noted in practice [44].

CONCLUSIONS

Whilst there may be an expectation that informal caregivers will provide support for people following hip fracture, our study illuminates challenges faced due to the acute and traumatic nature of hip fracture. We suggest that joint decision-making and goal-setting with health professionals, patients and their informal carers will enable better preparedness for the transition from hospital to home. This includes a greater explanation and tailoring of the recovery pathway especially for younger hip fracture patients.

For peer review only

ACKNOWLEDGEMENTS AND DECLARATIONS

Collaborators: The HIP HELPER Study Collaborators: Penny Clifford (Norfolk, PPI Representative), Lis Freeman (Norfolk, PPI Representative), Rene Gray (Principal Investigator – James Paget University Hospital NHS Trust), Yan Cunningham (Principal Investigator – City Hospitals Sunderland NHS Foundation Trust), Sarah Langford (Principal Investigator - Northumbria Healthcare NHS Foundation Trust), Dr Mark Baxter (Principal Investigator - University Hospital Southampton NHS Foundation Trust), Jessica Pawson – (Principal Investigator - Barts Health NHS Trust), Melissa Taylor (James Paget University Hospital NHS Trust), Anna Mellows (James Paget University Hospital NHS Trust), Kate Lacey (James Paget University Hospital NHS Trust), Alex Herring (City Hospital Sunderland NHS Foundation Trust), Diane Williams (Northumbria Healthcare NHS Foundation Trust), Anna Cromie (Northumbria Healthcare NHS Foundation Trust), Gail Menton (Northumbria Healthcare NHS Foundation Trust), Warren Corbett (University Hospital Southampton NHS Foundation Trust), Helen Jowett (University Hospital Southampton NHS Foundation Trust), Vishwanath Joshi (Barts Health NHS Trust), Maninderpal Matharu (Barts Health NHS Trust), Maria Baggot (University Hospital Southampton NHS Foundation Trust) and David Barker (University Hospital Southampton NHS Foundation Trust).

Oversight Committee Membership: TOC Members: Associate Professor Susan Dutton (Chair; University of Oxford, UK), Professor Opinder Sahota (University of Nottingham, UK), Dr Katie Sheehan (Kings College London, UK).

Authors' Contributors: SHa, AW, TS, SHo, KP, RK, PA, PL, MCo, SEL, MCr researched the topic and devised the study. TS, SHa, RK, PA, AC, SHo, AW, KP, PL, Mco, SEL, MCr provided the first draft of the manuscript. Sha lead the qualitative study design. AW lead the qualitative research conduct. TS, SHa, AW, RK, PA, AC, SHo, KP, PL, MCo, SEL, MCr contributed equally to manuscript preparation. TS acts a guarantor.

Funding Statement: This project is funded by the National Institute for Health and Care Research (NIHR) under its Research for Patient Benefit (RfPB) Programme (Grant Reference Number NIHR200731). MCo and SHo role in this study was supported by the National Institute for Health and Care Research Oxford Biomedical Research Centre. SEL role in this study was supported by the National Institute for Health and Care Research Exeter Biomedical Research Centre. The views expressed are those of the author(s) and not necessarily those of the NIHR or the Department of Health and Social Care.

Competing Interests Statement: None declared.

Disclaimer: MCo and SHo are supported by the National Institute for Health Research (NIHR) Oxford Biomedical Research Centre (BRC). SEL is supported by the National Institute for Health and Care Research Exeter Biomedical Research Centre. The views expressed are those of the author(s) and not necessarily those of the NHS, the NIHR or the Department of Health and Social Care.

Ethics Approval: North East - Newcastle & North Tyneside 1 Research Ethics Committee (20/NE/0213) Date: 16 March 2021.

Trial Sponsor: University of East Anglia (Research Sponsor, Research and Innovation Services, The Registry, University of East Anglia, Norwich, NR4 7TJ; Email: researchsponsor@uea.ac.uk) The views expressed are those of the author(s) and not necessarily those of the sponsor.

Provenance and Peer Review: Not commissioned; externally peer reviewed.

For peer review only

Data Sharing: The data that support the findings of this study may be available from the corresponding author (SH) upon reasonable request. This includes access to the full protocol, anonymised participant-level dataset and statistical code.

Patient consent for publication: Not required.

For peer review only

REFERENCES

1 Lee H, Cook JA, Lamb SE, *et al.* The findings of a surgical hip fracture trial were generalizable to the UK national hip fracture database. *J Clin Epidemiol* 2021;**131**:141-151. doi:10.1016/j.jclinepi.2020.11.016

2 Baker PN, Salar O, Ollivere BJ, *et al.* Evolution of the hip fracture population: Time to consider the future? A retrospective observational analysis. *BMJ Open* 2014;**4**:e004405. doi:10.1136/bmjopen-2013-004405

3 Lawler K, Taylor NF, Shields N. Involving family members in physiotherapy for older people transitioning from hospital to the community: A qualitative analysis. *Disabil Rehabil* 2015;**37**:2061-2069. doi:10.3109/09638288.2014.996673

4 Griffin XL, Parsons N, Achten J, *et al.* Recovery of health-related quality of life in a United Kingdom hip fracture population: The Warwick hip trauma evaluation - A prospective cohort study. *Bone and Joint Journal* 2015;**97-B**:372-382. doi:10.1302/0301-620X.97B3.35738

5 Seitz DP, Adunuri N, Gill SS, *et al.* Prevalence of Dementia and Cognitive Impairment Among Older Adults With Hip Fractures. *J Am Med Dir Assoc* 2011;**12**:556-564. doi:10.1016/j.jamda.2010.12.001

6 Em S, Bozkurt M, Caglayan M, *et al.* Psychological health of caregivers and association with functional status of stroke patients. *Top Stroke Rehabil* 2017;**24**:323-329. doi:10.1080/10749357.2017.1280901

7 Stajduhar KI, Funk L, Toye C, *et al.* Part 1: Home-based family caregiving at the end of life: A comprehensive review of published quantitative research (1998-2008). *Palliat Med* 2010;**24**:573-593. doi:10.1177/0269216310371412

8 Erickson ME. Effects of motivation, roles, coping strategies, and adaptations in relationships and personality on caretaking of elderly parents by midlife couples. Dissertation Abstracts International Section A: Humanities and Social Sciences. 2002;**62**.

9 Albertini M, Kohli M, Vogel C. Intergenerational transfers of time and money in European families: Common patterns - Different regimes? *J Eur Soc Policy* 2007;**17**:319-334. doi:10.1177/0958928707081068

10 Gérain P, Zech E. Informal Caregiver burnout? Development of a theoretical framework to understand the impact of caregiving. *Front Psychol* 2019;**10**. doi:10.3389/fpsyg.2019.01748

11 Feast A, Moniz-Cook E, Stoner C, *et al.* A systematic review of the relationship between behavioural and psychological symptoms (BPSD) and caregiver well-being. *Int Psychogeriatr*. 2016;**28**:1761-1774. doi:10.1017/S1041610216000922

12 Smith T, Clark L, Khoury R, *et al.* A feasibility study to assess the design of a multicentre randomized controlled trial of the clinical and cost-effectiveness of a caregiving intervention for people following hip fracture surgery. *Bone Jt Open* 2021;**2**:909-920. doi:10.1302/2633-1462.211.BJO-2021-0136

13 Skivington K, Matthews L, Simpson SA, *et al.* A new framework for developing and evaluating complex interventions: Update of Medical Research Council guidance. *The BMJ* 2021;**374**:n2061. doi:10.1136/bmj.n2061

14 Tong A, Sainsbury P, Craig J. Consolidated criteria for reporting qualitative research (COREQ): A 32-item checklist for interviews and focus groups. *Int J Qual Health Care* 2007;**19**:349-357. doi:10.1093/intqhc/mzm042

15 Ariza-Vega P, Ortiz-Piña M, Kristensen MT, *et al.* High perceived caregiver burden for relatives of patients following hip fracture surgery. *Disabil Rehabil* 2019;**41**:311-318. doi:10.1080/09638288.2017.1390612

16 Wimo A, Wetterholm AL, Mastey V, *et al.* Evaluation of the resource utilization and caregiver time in Anti-dementia drug trials—a quantitative battery. In: *The Health Economics of Dementia*. 1998.

For peer review only

17 Braun V, Clarke V. Using thematic analysis in psychology, *Qual Res Psychol* 2008;**3**:77-101. doi:10.1191/1478088706qp063oa

18 Boonsin S, Deenan A, Wacharasin C. Factors influencing the burden of family caregiving for survivors of stroke. *Pac Rim Int J Nurs Res Thail* 2020;**25**:102-113.

19 Ding TYG, de Roza JG, Chan CY, *et al.* Factors associated with family caregiver burden among frail older persons with multimorbidity. *BMC Geriatr* 2022;**22**:160. doi:10.1186/s12877-022-02858-2

20 Parry JA, Langford JR, Koval KJ. Caregivers of hip fracture patients: The forgotten victims? *Injury* 2019;**50**:2259-2262. doi:10.1016/j.injury.2019.09.030

21 Kim Y. Korean-American family post-caregivers on dementia caregiving: A phenomenological inquiry. *J Gerontol Soc Work* 2009;**52**. doi:10.1080/01634370903048352

22 Kuşçu MK, Dural U, Yaşa Y, *et al.* Decision pathways and individual motives in informal caregiving during cancer treatment in Turkey. *Eur J Cancer Care (Engl)* 2009;**18**:600-617. doi:10.1111/j.1365-2354.2007.00900.x

23 Øydgard GW. The influence of institutional discourses on the work of informal carers: An institutional ethnography from the perspective of informal carers. *BMC Health Serv Res* 2017;**17**:631. doi:10.1186/s12913-017-2591-7

24 Stajduhar KI, Martin WL, Barwich D, *et al.* Factors influencing family caregivers' ability to cope with providing end-of-life cancer care at home. *Cancer Nurs* 2008;**31**:77-85. doi:10.1097/01.NCC.0000305686.36637.b5

25 Zarzycki M, Morrison V, Bei E, *et al.* Cultural and societal motivations for being informal caregivers: a qualitative systematic review and meta-synthesis. *Health Psychol Rev* Published Online First: 2022;1-30. doi:10.1080/17437199.2022.2032259

26 Zarzycki M, Seddon D, Bei E, *et al.* Why do they care? A qualitative systematic review and meta-synthesis of personal and relational motivations for providing informal care. *Health Psychol Rev* 2022;1–33. doi:10.1080/17437199.2022.2058581

27 Griffiths F, Mason V, Boardman F, *et al.* Evaluating recovery following hip fracture: a qualitative interview study of what is important to patients. *BMJ Open* 2015;**5**:e005406–e005406. doi:10.1136/bmjopen-2014-005406

28 Cobley CS, Fisher RJ, Chouliara N, *et al.* A qualitative study exploring patients' and carers' experiences of Early Supported Discharge services after stroke. *Clin Rehabil* 2013;**27**:750-757. doi:10.1177/0269215512474030

29 Isik AT, Soysal P, Solmi M, *et al.* Bidirectional relationship between caregiver burden and neuropsychiatric symptoms in patients with Alzheimer's disease: A narrative review. *Int J Geriatr Psychiatry* 2019;**34**:1326-1334. doi:10.1002/gps.4965

30 Kuzuya M, Enoki H, Hasegawa J, *et al.* Impact of caregiver Burden on adverse health outcomes in community-dwelling dependent older care recipients. *Am J Geriat Psychiatry* 2011;**19**:382-391. doi:10.1097/JGP.0b013e3181e9b98d

31 Sutter-Leve R, Passint E, Ness D, *et al.* The caregiver experience after stroke in a covid-19 environment: A qualitative study in inpatient rehabilitation. *J Neurol Phys Ther* 2021;**45**:14-20. doi:10.1097/NPT.0000000000000336

32 Naylor MD, Shaid EC, Carpenter D, *et al.* Components of Comprehensive and Effective Transitional Care. *J Am Geriatr Soc* 2017;**65**:1119-1125. doi:10.1111/jgs.14782

33 Guilcher SJT, Everall AC, Cadel L, *et al.* A qualitative study exploring the lived experiences of deconditioning in hospital in Ontario, Canada. *BMC Geriatr* 2021;**21**:169. doi:10.1186/s12877-021-02111-2

34 Yuen EYN, Knight T, Ricciardelli LA, *et al.* Health literacy of caregivers of adult care recipients: A systematic scoping review. Health Soc Care Community. 2018;**26**:e191-e206. doi:10.1111/hsc.12368

For peer review only

35 Le DD, Ibuka Y. Understanding the effects of informal caregiving on health and well-being: Heterogeneity and mechanisms. *Soc Sci Med* 2023;**317**:115630. doi:10.1016/j.socscimed.2022.115630

36 Alpantaki K, Papadaki C, Raptis K, *et al.* Gender and Age Differences in Hip Fracture Types among Elderly: a Retrospective Cohort Study. *Maedica (Bucur)* 2020;**15**:185-190. doi:10.26574/maedica.2020.15.2.185

37 Bueno M, Chase J-A. Gender Differences in Caregiving of Older Adults: A Systematic Review of the Literature. *Innov Aging* 2020;**4**:150-151. doi:10.1093/geroni/igaa057.491

38 Fox F, Drew S, Gregson C, *et al.* Complex organisational factors influence multidisciplinary care for patients with hip fractures: a qualitative study of barriers and facilitators to service delivery. *BMC Musculoskelet Disord* 2023;**24**:128. doi:10.1186/s12891-023-06164-9

39 Magaziner J, Chiles N, Orwig D. Recovery after Hip Fracture: Interventions and Their Timing to Address Deficits and Desired Outcomes-Evidence from the Baltimore Hip Studies. *Nestle Nutr Inst Workshop Ser* 2015;**83**:71-81. doi:10.1159/000382064

40 Cameron JI, Gignac MAM. “Timing It Right”: A conceptual framework for addressing the support needs of family caregivers to stroke survivors from the hospital to the home. *Patient Educ Couns* 2008;**70**:305–14. doi:10.1016/j.pec.2007.10.020

41 Francis N, Hanna P. Informal carer experiences of UK dementia services—A systematic review. *J Psychiatr Ment Health Nurs*. 2022;**29**:116-129. doi:10.1111/jpm.12698

42 Fernández-Ballesteros R, Sánchez-Izquierdo M, Olmos R, *et al.* Cultural stereotypes in care contexts. *Clin Interv Aging* 2018;**13**:1613-1619. doi:10.2147/CIA.S169487

43 Dilworth-Anderson P, Williams IC, Gibson BE. Issues of race, ethnicity, and culture in caregiving research: A 20-year review (1980-2000). *Gerontologist*. 2002;**42**:237-272. doi:10.1093/geront/42.2.237

44 Veronese N, Maggi S. Epidemiology and social costs of hip fracture. *Injury* 2018;**49**:1458-1460. doi:10.1016/j.injury.2018.04.015

For peer review only

Table 1: Characteristics of sample interviewed

	Intervention Group N=7	Control Group N=3	Overall N=10
**Informal Caregiver**			
Age (years): median (IQR)	71.0 (58.0, 81.0)	71.0 (43.0, 72.0)	71.0 (58.0, 77.0)
Gender: n (%)			
- Male	5 (71.4%)	2 (66.7%)	7 (70.0%)
- Female	2 (28.6%)	1 (33.3%)	3 (30.0%)
Ethnicity: n (%)			
- White British	6 (85.7%)	3 (100%)	9 (90.0%)
- White other	1 (14.3%)	0	1 (10.0%)
Relationship to person with hip fracture: n (%)			
- Spouse	4 (57.1%)	2 (66.7%)	6 (60.0%)
- Daughter/son	1 (14.3%)	1 (33.3%)	2 (20.0%)
- Other	2 (28.6%)	0	2 (20.0%)
Employment: n (%)			
- Not working	6 (85.7%)	3 (100%)	9 (90.0%)
- Part-time work	1 (14.3%)	0	1 (10.0%)
**Person with hip fracture**			
Age at consent (years): median (IQR)	79.0 (70.0, 82.0)	69.0 (68.0, 71.0)	72.5 (69.0, 79.0)
Gender: n (%)			
- Male	1 (14.3%)	0	1 (10.0%)
- Female	6 (85.7%)	3 (100%)	9 (90.0%)
Ethnicity: n (%)			
- White British	7 (100%)	3 (100%)	10 (100%)
Has Cognitive Impairment (based on AMTS category): n (%)	1 (14.3%)	0	1 (10.0%)
AMTS score at consent: median (IQR)	10.0 (9.0, 10.0)	10.0 (10.0, 10.0)	10.0 (9.0, 10.0)
NEADL score at baseline: median (IQR)	20.0 (14.0, 22.0)	10.0 (10.0, 10.0)	17.0 (12.0, 22.0)

**Location**		
Site (n)		
1	1	1
2	1	1
3	1	1
4	3	-
5	1	-

Abbreviations

AMTS Abbreviated Mental Test Score
NEADL Nottingham Extended Activities of Daily Living Scale

Topic	Sample questions	Prompts/probes
Overall	• Overall, do share your experiences of being involved with our research?	
The approach and consent process	• Please talk me through how you got involved with the HipHelper research study?<br>• How clearly was the study explained to you?	• Could we have dealt with that differently?<br>• How was this different to what you expected?<br>• For the future, what could we improve about the description of the study?
Willingness to be randomised to either group/ participant views of their intervention<br><br>The risk of intervention contamination between the groups	• Please talk me through what study treatment you received<br><br>• How much did talk to any other patients or caregivers (whilst in hospital) about the intervention?	• *If required: clarify what HIP HELPER was and what was usual care/non-study intervention*<br>• You were allocated to X group. What did that feel like?<br>• What discussions were had between those who received it and did not receive it?
**Both groups**		
The acceptability of the inpatient care	• Please talk me through your treatment while you were in the hospital	• What aspects of your care that were lacking in the hospital?<br>• Can you remember which wards you were on? Can you remember being moved to different areas of the hospital?

For peer review only

**Topic**	**Sample questions**	**Prompts/probes**
	• *(Carers)* As X's carer, what was your impression of the care?	• For both of you, what was helpful and less helpful to your care?
**Intervention group only**		
In-patient HIP HELPER programme<br><br>Strengths/weaknesses	• How did you find the HIP HELPER programme?	• Please share any specific examples of what you can remember from the programme?<br>• What were the most helpful bits of your HIP HELPER intervention?<br>• What was good about it?<br>• What were the less helpful/worse bits of the HIP HELPER intervention?<br>• For you as the patient/and you as X's carer?
	• Please tell me what you remember about the manual handling training you had in the hospital?	• How did you get on with that at home?<br>• Was it helpful? If so, how?
	• Please tell me about the phone calls you received?	• Can you remember what you talked about?<br>• Can you give specific examples of what was helpful?<br>• Was there any advice that confused you or you weren't clear about?
	• What were the individualised goals you set in the hospital?	• How did you come up with these?<br>• *(Carer)* By how far were you involved in the goal setting process?<br>• In retrospect, what goal(s) would you alter?

For peer review only

Topic	Sample questions	Prompts/probes
**Both groups:**		
On return to your home	• *(If required, state residential status)* Can you tell me who you live with at home?	
	• What were the first things you wanted to achieve once at home?<br>• How did you decide who was to be your allocated carer?<br>• *(Carers)* Can you talk me through this decision to be designated as X's carer?	• On a 1-10 scale, how confident did you feel when you left the hospital?<br>• Can you tell me why you have chosen that number on the scale?
	• *(Long-term carers)* Once at home, how do you feel care-giving role changed?	• Can you tell me about any new caring responsibilities?<br>• How did you organise these?<br>• Did this change the dynamics in their relationship in taking on caring role, if so, how
	• What sort of adaptions did you make at home to enable you to move about independently (where possible)?<br>• What do you think is lacking in the transition from hospital to home?	• Are/were there any other things that may have affected your transfer home?<br>• Are/were there any other things that required you to adapt the advice you were given?
	• How willing were/are you to continue with activities/advice that were suggested to you?<br>• Once at home, by how far did you feel supported by the health care professions you had seen?	*Probes: transferring, dressing, activities of daily living*

For peer review only

Topic	Sample questions	Prompts/probes
	• *(Carers)* For you as caregiver, how did you get on with helping move X about?<br>• Were there any adaptions that you made to the advice given?	
What modifications they may recommend to interventions received	• What changes did you find easy to implement and what did you find harder?	• What could we improve?
	• What do we need to modify for any future programmes to support people with a hip replacement and their carer to help you get up and moving and returning to normal life?	• Can you explain why? *Prompt around ADL's*
The ease and convenience of the data collection processes/applicability of the methods and measures used	• As you were part of a trial, we had to collect a lot of measurements. Can you talk me through what these were?	*Prompt:*<br>*'Share screen' of the front page of questionnaires to help with memory recall.*
	• How did you manage with the questionnaires we gave you at the start of the study and at the end in the post?<br>• What about your interview experience today?	• Were they easy to complete?<br>• Do you remember them being a problem?<br>• How convenient were they?<br>• How did you find it?<br>• Is there anything we can do to improve this experience?
Summary and end	• We are now ? months since your hip replacement, by how far do you think the HipHelper programme (*or usual care)* has helped with your recovery?<br>• If we were to run this programme again in the future, is there anything else we should consider?	• How far do you think the Hip Helper programme (*or usual care)* has helped you achieve your goals?

For peer review only

Topic	Sample questions	Prompts/probes
	• How do you think we could better support you and your carer to recover after hip surgery?<br>• Is anything else you have gained by being involved in this research study<br>• Is there anything else we haven't thought of and that you can share with us to improve the study and the care of hip replacement patients and their carers?	

For peer review only

COREQ (COnsolidated criteria for REporting Qualitative research) Checklist

A checklist of items that should be included in reports of qualitative research. You must report the page number in your manuscript where you consider each of the items listed in this checklist. If you have not included this information, either revise your manuscript accordingly before submitting or note N/A.

Topic	Item No.	Guide Questions/Description	Reported on Page No.
**Domain 1: Research team and reflexivity**			
Personal characteristics			
Interviewer/facilitator	1	Which author/s conducted the interview or focus group?	
Credentials	2	What were the researcher's credentials? E.g. PhD, MD	
Occupation	3	What was their occupation at the time of the study?	
Gender	4	Was the researcher male or female?	
Experience and training	5	What experience or training did the researcher have?	
Relationship with participants			
Relationship established	6	Was a relationship established prior to study commencement?	
Participant knowledge of the interviewer	7	What did the participants know about the researcher? e.g. personal goals, reasons for doing the research	
Interviewer characteristics	8	What characteristics were reported about the inter viewer/facilitator? e.g. Bias, assumptions, reasons and interests in the research topic	
**Domain 2: Study design**			
Theoretical framework			
Methodological orientation and Theory	9	What methodological orientation was stated to underpin the study? e.g. grounded theory, discourse analysis, ethnography, phenomenology, content analysis	
Participant selection			
Sampling	10	How were participants selected? e.g. purposive, convenience, consecutive, snowball	
Method of approach	11	How were participants approached? e.g. face-to-face, telephone, mail, email	
Sample size	12	How many participants were in the study?	
Non-participation	13	How many people refused to participate or dropped out? Reasons?	
Setting			
Setting of data collection	14	Where was the data collected? e.g. home, clinic, workplace	
Presence of non-participants	15	Was anyone else present besides the participants and researchers?	
Description of sample	16	What are the important characteristics of the sample? e.g. demographic data, date	
Data collection			
Interview guide	17	Were questions, prompts, guides provided by the authors? Was it pilot tested?	
Repeat interviews	18	Were repeat inter views carried out? If yes, how many?	
Audio/visual recording	19	Did the research use audio or visual recording to collect the data?	
Field notes	20	Were field notes made during and/or after the inter view or focus group?	
Duration	21	What was the duration of the inter views or focus group?	
Data saturation	22	Was data saturation discussed?	
Transcripts returned	23	Were transcripts returned to participants for comment and/or	

For peer review only

Topic	Item No.	Guide Questions/Description	Reported on Page No.
		correction?	
**Domain 3: analysis and findings**			
Data analysis			
Number of data coders	24	How many data coders coded the data?	
Description of the coding tree	25	Did authors provide a description of the coding tree?	
Derivation of themes	26	Were themes identified in advance or derived from the data?	
Software	27	What software, if applicable, was used to manage the data?	
Participant checking	28	Did participants provide feedback on the findings?	
Reporting			
Quotations presented	29	Were participant quotations presented to illustrate the themes/findings? Was each quotation identified? e.g. participant number	
Data and findings consistent	30	Was there consistency between the data presented and the findings?	
Clarity of major themes	31	Were major themes clearly presented in the findings?	
Clarity of minor themes	32	Is there a description of diverse cases or discussion of minor themes?	

Developed from: Tong A, Sainsbury P, Craig J. Consolidated criteria for reporting qualitative research (COREQ): a 32-item checklist for interviews and focus groups. *International Journal for Quality in Health Care*. 2007. Volume 19, Number 6: pp. 349 – 357

Once you have completed this checklist, please save a copy and upload it as part of your submission. DO NOT include this checklist as part of the main manuscript document. It must be uploaded as a separate file.

For peer review only

BMJ Open

Perspectives of informal caregivers who support people following hip fracture surgery: a qualitative study embedded within the HIP HELPER feasibility trial.

Journal:	*BMJ Open*
Manuscript ID	bmjopen-2023-074095.R1
Article Type:	Original research
Date Submitted by the Author:	12-Sep-2023
Complete List of Authors:	Welsh, Allie; University of East Anglia, School of Education<br>Hanson, Sarah; University of East Anglia, School of Health Sciences<br>Pfeiffer, K; Robert Bosch Hospital, Department of Clinical Gerontology and Geriatric Rehabilitation<br>Khoury, Reema; University of East Anglia,<br>Clark, Allan; University of East Anglia, Norwich Medical School<br>Ashford, Polly-Anna; University of East Anglia, Norwich Clinical Trials Unit<br>Hopewell, Sally; University of Oxford, Nuffield Department of Orthopaedics, Rheumatology and Musculoskeletal Sciences<br>Logan, Phillipa; University of Nottingham, Community Health Sciences<br>Crotty, Maria; Flinders University, Rehabilitation, Aged and Extended Care<br>Costa, Matthew; University of Oxford, Nuffield Department of Orthopaedics, Rheumatology, and Musculoskeletal Sciences<br>Lamb, Sarah; University of Exeter, College of Medicine and Health<br>Smith, Toby; University of Warwick; University of East Anglia, School of Health Sciences
**Primary Subject Heading**:	Surgery
Secondary Subject Heading:	Qualitative research, Rehabilitation medicine
Keywords:	Hip < ORTHOPAEDIC & TRAUMA SURGERY, QUALITATIVE RESEARCH, REHABILITATION MEDICINE

SCHOLARONE™
Manuscripts

I, the Submitting Author has the right to grant and does grant on behalf of all authors of the Work (as defined in the below author licence), an exclusive licence and/or a non-exclusive licence for contributions from authors who are: i) UK Crown employees; ii) where BMJ has agreed a CC-BY licence shall apply, and/or iii) in accordance with the terms applicable for US Federal Government officers or employees acting as part of their official duties; on a worldwide, perpetual, irrevocable, royalty-free basis to BMJ Publishing Group Ltd ("BMJ") its licensees and where the relevant Journal is co-owned by BMJ to the co-owners of the Journal, to publish the Work in this journal and any other BMJ products and to exploit all rights, as set out in our licence.

The Submitting Author accepts and understands that any supply made under these terms is made by BMJ to the Submitting Author unless you are acting as an employee on behalf of your employer or a postgraduate student of an affiliated institution which is paying any applicable article publishing charge ("APC") for Open Access articles. Where the Submitting Author wishes to make the Work available on an Open Access basis (and intends to pay the relevant APC), the terms of reuse of such Open Access shall be governed by a Creative Commons licence – details of these licences and which Creative Commons licence will apply to this Work are set out in our licence referred to above.

Other than as permitted in any relevant BMJ Author's Self Archiving Policies, I confirm this Work has not been accepted for publication elsewhere, is not being considered for publication elsewhere and does not duplicate material already published. I confirm all authors consent to publication of this Work and authorise the granting of this licence.

For peer review only

BMJ OPEN

TITLE PAGE

Title: Perspectives of informal caregivers who support people following hip fracture surgery: a qualitative study embedded within the HIP HELPER feasibility trial

Authors: Welsh A,[1] Hanson S,[2] Pfeiffer K,[3] Khoury R,[2] Clark A,[2] Ashford P-A,[2] Hopewell S,[4] Logan PA,[5] Crotty M,[6] Costa ML,[4] Lamb SE,[7] Smith TO[2,8] on behalf of the HIP HELPER Study Collaborators.

Affiliations

1. School of Education, University of East Anglia, Norwich, UK
2. Faculty of Medicine and Health Sciences, University of East Anglia, Norwich, UK
3. Department of Geriatric Rehabilitation, Robert-Bosch-Hospital, Stuttgart, Germany
4. Nuffield Department of Rheumatology, Orthopaedics and Musculoskeletal Sciences, University of Oxford, Oxford, UK
5. School of Medicine, University of Nottingham, Nottingham, UK
6. College of Medicine and Public Health, Flinders University, Adelaide, Australia
7. College of Medicine and Health Sciences, University of Exeter, Exeter, UK
8. Warwick Medical School, University of Warwick, Coventry, UK

Corresponding Author: Dr Allie Welsh, School of Education, University of East Anglia, Norwich, NR4 7TJ. Email: a.welsh@uea.ac.uk

For peer review only - http://bmjopen.bmj.com/site/about/guidelines.xhtml

ABSTRACT

Objectives: To illuminate the perspectives of informal caregivers who support people following hip fracture surgery.

Design: A qualitative study embedded within a now completed multi-centre, feasibility randomised controlled trial (HIPHELPER).

Setting: Five English National Health Service (NHS) hospitals.

Participants: We interviewed 20 participants (10 informal caregivers; 10 people with hip fracture), following hip fracture surgery. This included one male and nine females who experienced a hip fracture; and seven male and three female informal caregivers. The median age was 72.5 years (range: 65 to 96 years), 71.0 years (range: 43 to 81 years) for people with hip fracture and informal caregivers, respectively.

Methods: Semi-structured, virtual interviews were undertaken between November 2021 and March 2022, with caregiver dyads (person with hip fracture and their informal caregiver). Data were analysed thematically.

Findings: We identified two main themes: expectations of the informal caregiver role, and reality of being an informal caregiver; and sub-themes: expectations of care and services; responsibility and advocacy; profile of people with hip fracture; decision to be a caregiver; transition from hospital to home.

Conclusion: Findings suggest informal caregivers do not feel empowered to advocate for a person's recovery or navigate the care system, leading to increased and unnecessary stress, anxiety and frustration when supporting the person with hip fracture. We suggest that a tailored information-giving on the recovery pathway, which is responsive to the caregiving population (i.e., considering the needs of male, younger, and more active informal caregivers and people with hip fracture) would smooth the transition from hospital to home.

Key words: informal caregivers; hip fracture; recovery; rehabilitation; carer; qualitative

Trial registration number: ISRCTN13270387

For peer review only

For peer review only - http://bmjopen.bmj.com/site/about/guidelines.xhtml

STRENGTHS AND LIMITATIONS OF THIS STUDY

- We describe the contextual factors of participating in HIP HELPER and people's experiences of giving and receiving informal care.
- This study may help health professionals support informal caregiver dyads in the transition from hospital to home.
- Participants presented with limited socioeconomic, cultural or ethic perspectives, and there was a lack of perspectives of those caring for a person with cognitive impairment.
- COVID-19 pandemic affected NHS services which may have impacted on the study delivery and participant's experiences.

Word Count: Manuscript: 4214; Abstract: 252

INTRODUCTION

Hip fracture is a devastating injury, predominantly seen in frail older people, and disproportionally more women [1]. Approximately 80,000 people aged 60 years and over experience a hip fracture in the UK each year, resulting in a combined health and social cost of at least £2 billion [2]. This cost is expected to increase with an ageing population [1]. Outcomes following hip fracture are poor. The majority of individuals do not return to pre-injury levels of function, and frequently lose independence and self-caring abilities [3,4].

Given the sudden, traumatic nature of hip fracture, people often rely on family members or friends for informal care following hospital discharge. Additionally, approximately 40% of people who sustain a hip fracture have cognitive impairment, meaning many caregivers also have to cope with the challenges of functional dependency and neurocognitive symptoms [5]. These 'informal caregivers' therefore play an integral role, in the transition from hospital to home, the initial discharge period and possibly beyond [6].

Informal caregivers are a heterogeneous population. For some, this may be the first time with caregiving responsibilities; for others, it is perceived as an episodic event with a temporary increase in care needs. They may be a retired spouse, friend, grown-up children in employment; living with, close by or far away; and of course, they be facing their own physical and mental health challenges. Caregiving may further be exacerbated by (but not limited to) the ageing population, smaller average family size, changing gender-roles due in part to more women working outside the home [7–9].

The ability of a person to cope with the roles and responsibility of caregiving is a complex interaction of different factors. In addition to stressors related to the actual caregiving, there are other factors that may determine a person's ability to manage the caregiving role, such as: caregiver resources, social environment (e.g., informal and professional support), relationship quality with the care-recipient and caregiver, positive aspects of caregiving (e.g., finding meaning in the caregiving role) [10]. Whilst caregiving experiences have been reported in other populations such as stroke [6,11] and dementia [12], there is still much to understand about acute episodes of informal care, such as in hip fracture. Previous literature suggests that the experiences of recovery after hip fracture for patients and informal caregivers may be different to these previous populations [13]. These may include differing recovery trajectories and impairment [14], greater challenges with post-operative pain management, nausea and fear avoidance [15].

For peer review only - http://bmjopen.bmj.com/site/about/guidelines.xhtml

Our qualitative study investigated the informal caregiver perspectives of caring for a person with hip fracture following hospital discharge, nested within a larger study aimed at assessing the feasibility of a pragmatic, multi-centre randomised controlled trial (RCT) of an informal caregiver training programme (HIP HELPER) to support the recovery of people following hip fracture surgery [16].

METHODS

This was a qualitative study embedded within a multi-centre, feasibility randomised controlled trial (HIP HELPER). The HIP HELPER protocol is published elsewhere, and the study is now complete [16]. In brief, HIP HELPER aimed to assess the design of a pragmatic RCT to test the effectiveness of an informal caregiver intervention, compared to usual NHS care following hip fracture surgery. Caregiver dyads who were randomised to receive the HIP HELPER intervention were allocated to received three, one- hour, one-to-one training sessions, delivered by either, a nurse, a physiotherapist, or an occupational therapist. Training sessions included practical skills for rehabilitation such as: transfers and walking, pacing, and stress management techniques, and the provision of and the HIP HELPER Caregiver Workbook, offered information on recovery, exercises, worksheets, and goal-setting plans to facilitate a 'good' recovery.

The present study reports the experiences and contextual factors of undergoing hip fracture surgery and dyad's experiences of giving and receiving informal care. Patient and caregiver perspective of the experimental caregiving training programme are to be reported with the feasibility study results. Group allocation (intervention or control) was therefore not a significant consideration for the present study. We followed the COREQ reporting guidelines [17].

Fourteen caregiver dyads were purposively sampled by age, pre-fracture disability, and hospital locations. Patients were those who had undergone hip fracture surgery. An informal caregiver was defined as someone who was expected to informally provide care, assistance, support or supervision in activities of daily living (ADLs) for at least three hours per week, but not on a paid basis. This may have included personal ADLs such as toileting, washing, dressing and eating; and/or more complex tasks such as managing money, shopping and household chores [18,19]. Other inclusion criteria for participants were men and women 60 years and above, community-dwelling prior to admission, able to attend face-to-face hospital appointments and/or access to a computer/table and internet services to receive a video consultation call.

Data Collection

In-depth, semi-structured interviews were undertaken with caregiver dyads, within six-weeks of hospital discharge, by AW, who is a white, female post-doctoral researcher. AW had no role in recruitment to the study nor intervention delivery, thus was not known to participants. Interviews were conducted virtually using Microsoft Teams or via telephone, between November 2021 and March 2022. They were audio-recorded, transcribed and all identifying information removed.

It was not necessary to operationalise the concept of saturation, firstly given our sampling approach, and secondly given we recognise that meaning is generated through interpretation of data are inescapably situated and subjective [20,21]. As per Hanson et al's [22] approach, we analysed the data to identify recurrent themes generated from the analysis. At 10 participant-dyads, we started to see recurring themes and ideas. Accordingly, we made a pragmatic decision that we had collected sufficient data to be representative of the hip fracture informal caregiver dyad experiences and ceased data collection.

For peer review only - http://bmjopen.bmj.com/site/about/guidelines.xhtml

Data Analysis

Using the principles of thematic analysis, data were initially analysed deductively against our questioning framework and then further explored inductively to explore contextual features and participants experiences [23]. AW generated initial codes and themes. AW, SH, TS and KP then engaged in discussion to develop, review, refine and name themes.

Reflexivity was acknowledged in the design and analysis of our study. This included reflecting on the positions of researcher team (TS – physiotherapist, SHa – nurse, AW – physiologist, KP - psychologist) to ensure we understood the context and significance were interpreted appropriately and our predetermined positions were appreciated [24,25].

We did not return our transcripts to participants for comment or correction, and we did not seek ethical approval to do so. This is based on our experience as qualitative researchers and the little evidence available that suggests member checks improve research findings [26,27]. Participants were thus not involved in the data analysis process for these reasons and the lack of resources available for training individuals.

Patient and Public Involvement (PPI)

Patient involvement began during protocol development and continued throughout the trial. The patient-member was a co-investigator, who provided insights into the trial conduct and supported the interpretation of findings during the trial's dissemination phase.

The topic guide was developed with two members of the public, who helped guide the approach and style of the interview topic guides through a pilot interview (**Supplementary File 1**). Learnings from the pilot included some rewording of questions for clarity and the use of prompts to further unpick experiences. As suggested by PPI members, the topic guide was shared with participants prior to the interview, enabling them to reflect and plan beforehand.

FINDINGS

Participants

In total, 10 interviews were undertaken with caregiver dyads (20 participants) across our five study sites in England. One dyad declined due to other commitments, one did not give a reason and the remaining dyads did not take part in the interview as the person with hip fracture had been readmitted to hospital. The median length of interviews was 33 minutes (range: 27 to 53 minutes).

Dyad characteristics are presented in **Table 1**. The median age of people with hip fracture and their caregiver was 72.5 years (range: 65 to 96 years) and 71.0 years (range: 43 to 81 years), respectively. Of the informal caregivers, seven were male and three were female. There were three males and seven females who experienced a hip fracture. Overall, six informal caregivers were the spouse of the person with hip fracture, two were adult children (a daughter and a son) and two were described as 'other'.

For peer review only - http://bmjopen.bmj.com/site/about/guidelines.xhtml

Themes

Overall, two main themes and associated sub-themes were identified:

(1) Expectations of the informal caregiver role
  a) Expectations of care and services
  b) Responsibility and advocacy

(2) Reality of being an informal caregiver
  a) Profile of people with hip fracture
  b) Decision to be a caregiver
  c) Transition from hospital to home

Expectations of the informal caregiver role

Informal caregivers perceived that health professionals assumed that they would immediately take-on (additional) caregiving responsibilities upon the discharge home.

"Assumed that because I'm working from home [I would care], just said it like, didn't even ask me." (Caregiver, Female)

a) Expectations of care and services

Both informal caregivers and people with hip fracture suggested that rather than being personalised, goals were set by health professionals on a 'standard' patient recovery trajectory and therefore these were not viewed as achievable, nor realistic. They also had insufficient information to understand what a typical recovery trajectory looks like. It appears that most people did not feel empowered and found goal setting and developing realistic recovery goals difficult, based on imperfect information.

"I had no expectations because I have never been in hospital before. Ever. I have never been ill before, so this is completely new to me and obviously new to [caregiver] – he's never had to care for me before." (Person with Hip Fracture, Female)

"Not quite enough available to you about kind of next steps and what's going to happen in the future." (Caregiver, Male)

In contrast, again for informal caregivers and people with hip fracture, receiving knowledge on recovery expectations with realistic goal-setting and joint decision-making, as seen in our HIP HELPER intervention, appeared to be empowering.

"If you get somebody who doesn't have sufficiently stimulating support, that is a problem because in a sense, it encourages them to stay dependent, whereas what it should be doing is encourage them to become independent." (Caregiver, Male)

b) Responsibility and advocacy

Caregivers reported a sense that they must advocate and even battle for services to achieve adequate care and support in the transition from hospital to home, and yet, they had no power to do so.

For peer review only - http://bmjopen.bmj.com/site/about/guidelines.xhtml

Navigating the care system is difficult and requires knowledge and confidence. This was particularly pertinent among first-time caregivers.

"There is only as much as I can do to protect my husband. He's got a right to have a minimum care. Really, we're not asking for much." (Caregiver, Female)

"I had to make phone calls and chase around to try and sort things out [for her recovery at home]." (Caregiver, Female)

Informal caregivers felt that this level of advocacy was time consuming and created an imbalance in undertaking care activities, thus detracting from giving personal support.

"But while I have to chase up all these things, he's not getting the best. He needs my attention, you know, to make sure that he's doing his exercise." (Caregiver, Female)

Caregivers also expressed their feelings of accountability for supporting independence and recovery and, not surprisingly for some, this induced feelings of stress and anxiety.

"She's always been very independent, and I just felt very responsible for it. Seems to be quite a lot on my shoulders." (Caregiver, Male)

"There was a shock when she fell and broke her hip. It's a lot to take on. All of a sudden, everything falls to one person, it's tough." (Caregiver, Male)

<u>Reality of being an informal caregiver</u>

There was a dissonance in the expectations of being a caregiver and the actual role. The reality was shaped by the motivation and willingness to become a caregiver, the profile of the caregiver and the person with hip fracture, and the process of being discharged home. This mismatch was seen more in first-time caregivers and spouses of the person with hip fracture.

a) Profile of the caregiver and the person with hip fracture

The majority (n=8/10) of caregivers were first-time caregivers with little or no understanding of the hip fracture recovery process.

"His wife had Alzheimer's and he cared for her for about 16 years. So, it's something that he understands. And he, well, he's just very good at it." (Person with Hip Fracture, Female)

For those caregivers in full-time employment, competing priorities between work and caring highlighted the reality of being an informal caregiver.

"I had loads of work on and I was having to work at 10 o'clock at night to try and get my work done, because through the day when places were open, I was fooling around trying to get the things we need, sort mam and do her appointments." (Caregiver, Female)

The profile of people with hip fracture in this study, were generally active older adults, some of whom were still in employment and felt the services available post-hip fracture, were not conducive to their lifestyle, needs or goals. They perceived services are primarily designed for older adults, suggesting

For peer review only - http://bmjopen.bmj.com/site/about/guidelines.xhtml

inadequacy in rehabilitation for those with hip fracture (and their caregivers) aiming to return to occupational or physically demanding leisure activities.

> *"I don't think we're typical patients and carers. Certainly, I am definitely not a typical carer, and *** is definitely not a typical patient."* (Caregiver, Male)

> *"You know, I'm not a little old lady who sits and knits in the chair and doesn't move about much so that makes a difference, doesn't it?"* (Person with Hip Fracture, Female)

Caregivers were also mindful of their own age and health status. Caregivers were aware that they required physical and mental capacity to effectively care for the person following hip fracture and that maintaining their own health and wellbeing was equally as important.

> *"I was conscious that I had to take care of myself to be fit to be looking after her." (*Caregiver, Male)

> *"You know I'm so lucky that I'm healthy still. That I'm young and capable, but, who knows, you know?"* (Caregiver, Male)

Caregivers recognised the need to balance their own physical and psychological needs with their care-recipient's need.

b) Decision to be a caregiver

Findings identified multiple factors which contributed to the decision to become an informal caregiver. Spousal obligation and acting out of affection, were examples identified from the present study. For some, values such as loyalty and commitment arising from marriage (spousal obligation) was what motivated people to take on a caregiving.

> *"We both met two and a half years ago. Dare I say it nuts about each other? Ever since then, we've been living together, as man and wife."* (Caregiver, Male)

For others, there was the absence of *actual choice*, in-part attributed to assumptions made on discharge planning, but also underpinned by the unavailability of alternative care options.

> *"I think in our case there wouldn't have been anyone else who would be able to come and help."* (Caregiver, Male)

An interesting point about the decision to be a caregiver is that no participant expressed an awareness of caring for a limited acute period of time. Thus, it is unknown how long caregivers perceive the responsibility of caring for a person with hip fracture lasts for.

c) Transition from hospital to home

Caregivers expressed frustration, confusion, and uncertainty at the point of transitioning home. Such feelings were principally attributed to a lack of communication throughout discharge planning with caregivers feeling isolated, under-prepared and excluded from the decision-making. Visitor restrictions due to the COVID-19 pandemic, may have also reduced communication channels and contributed to caregiver uncertainty, resulting in concerns of whether the person with hip fracture was receiving adequate.

For peer review only - http://bmjopen.bmj.com/site/about/guidelines.xhtml

"It's very difficult. I was at one point sending her back to hospital 'cause I was so frantic with it all." (Caregiver, Female)

"Nothing is said [about discharge] at the hospital, apart from telling you have an appointment in eight weeks, there's nothing else said." (Caregiver, Male)

"I mean the one thing that I do remember is really actually a feeling of isolation and a little bit concerned at whether or not I was doing the right things to support her." (Caregiver, Male)

The profile of caregivers also contributes to their competence in managing the transition home, potentially relying on previous experiences to navigate the health system and available services.

"You know, imagine if you if you weren't an old nurse like me? How on earth can you even navigate the system? I think people are getting lost out there and it's creating a lot of problems for the health service." (Caregiver, Female)

Some participants shared how they had to 'beg, steal and borrow' equipment from friends and family. It is apparent that the onus is on the caregiver themselves to navigate and seek the appropriate provisions for recovering at home with hip fracture and there was a feeling of abandonment.

"Luckily, some family had a spare single bed. A friend had a perching stool and a trolley. And then another friend, sadly her husband had been ill, but they had purchased their own wheelchair and we actually borrowed that wheelchair. And a cousin, he built some ramps so I could get the wheelchair in." (Caregiver, Female)

DISCUSSION

Previous studies have provided important insights into the experiences of patients and caregivers following hip fracture, which we have further explored to better understand this challenge [13,28]. Our study aimed to understand the perspectives of informal caregivers who support the recovery of people following hip fracture surgery. Findings indicate that there is a tension between the expectations, and reality of the caregiving role for people with hip fracture. This is largely attributed to caregivers feeling disempowered to advocate for a person's recovery, leading to increased stress, anxiety and frustration when supporting them. Additionally, caregivers struggle with 'juggling' their own life with their caregiving role (e.g., full-time employment [29], whilst also maintaining their own health and well-being, for the benefit of both members of the dyad when discharged home [30]. There is a growing body of literature suggesting the mental health and wellness of a caregiver is also linked with a patient's functional and health outcomes [30–32]. Maintaining physical and mental ability to care for an individual is therefore important for hip fracture recovery.

This study identified that discharge processes after hip fracture do not currently fully prepare informal caregivers for their roles. As such, key learning points for health professionals are suggested to improve collaboration and support in the transition from hospital to home. These include: a greater explanation on 'normal' recovery processes; engaging for (dyad) joint decision-making; and promote the provision of additional (community) services for support. This is supported by previous findings that have also highlighted several key recommendations for translational care which emphasise dyad engagement, education, and wellbeing [33,34].

For peer review only - http://bmjopen.bmj.com/site/about/guidelines.xhtml

Joint decision-making based on setting realistic and tailored goals, is another practical strategy health professionals may employ to support the transition of people with hip fracture and their informal caregiver from hospital to home. This agrees with previous authors such as Angeli et al [35] and Brewer et al [36] and re-enforced in Saletti-Cuesta et al's [13] systematic review, who stressed that collaborative goal setting can allow designated time to enhance family involvement, to focus on functional outcomes and highlight current priorities for the caregiving dyad. It therefore may be that informal caregiver are well-placed to act as a 'driver of goal-setting', as they may have intimate knowledge of the person pre-hip fracture (for example, preferences and character).

A key finding of this study was the powerlessness which caregivers felt they had in supporting and advocating for the person with hip fracture. As a result of their inability to advocate for the person with hip fracture, participants frequently reported feelings of either over anticipation (negative) contrasted with unrealistic (positive) opinions on what could be offered and provided to the person who they were supported. This is a common theme that resonates within previous literature [18,37,38]. Tutton et al [28] acknowledged the uncertainties shared by patients and caregivers after hip fractures and change of relationship within the caregiving dyad through disability. There was an expectation by health professionals did not sufficiently appreciate this uncertainty and particularly following a change in health status. Our findings suggest that health professionals expected that caregivers could navigate the health and social care system, which would indeed, require some level of health literacy. It is known that low health literacy among caregivers has the potential to impact adequate care provision [39]. This is particularly pertinent among those who are 'new' caregivers, those who may perceive to be particularly under-skilled, under-prepared and/or be adjusting to a new role or identify as an informal caregiver. This is contrary to experienced caregiver who could elicit competence, knowledge, and tolerance in caregiving [40–43]. Additionally, the profile of a caregiver may include a natural affinity to caring, namely a 'caring nature' [29,44]. There is therefore the need to develop interventions that improve informal caregiver health literacy, ultimately upskilling informal caregivers to have the confidence to advocating for the person with hip fracture.

This study offers insights into an informal caregiver population which may differ to most previously reported. Previous literature has emphasised the caregiver experience of caring for people with long-term conditions which is unlikely to be transferable to the acute, traumatic, and rapid nature of caregiving for people with hip fracture [45]. This study therefore offers unique perspectives on a different transition in becoming a (informal) caregiver, particularly as the findings suggest little time for decision-making and the assumption of accepting this role of significant responsibility [46]. A second element to this caregiving population is how it is gendered. Caregiving is known to have a greater adverse effect on female caregivers' mental health and life satisfaction [47]. However, the demographic of hip fracture is largely female (considering experiences of the menopause and osteoporosis, to name a few), as seen in our larger study necessitating male partners to take on the caring role who may find undertaking sensitive tasks more challenging [45,48]. Additionally, the qualities and skills frequently offered by female caregivers previously reported are that females are better communicators, better instigators of help and are better organised, suggesting that male caregivers might benefit with tailored support for these potential challenges [44]. Whilst it is recognised that expertise in managing frail, older patients is crucial to ensure holistic care for hip fracture patients [49], our study also featured much younger hip fracture participants, who had quite different perceptions of what was important to them and expectations of recovery [29,50].

This study presents with strengths and limitations. A notable strength was its ability to capture experiences during a novel time in healthcare history, where hospitals were making rapid, visitor policy changes in response to the COVID-19 pandemic. Due to the timing of the study, the participants shared a distinctive experience and its effect on rehabilitation and on the caregiver experience. However, one may also interpret this as weakness as it is not a typical reflection of care for this

For peer review only - http://bmjopen.bmj.com/site/about/guidelines.xhtml

population. Interviews took place within four-months post-hip fracture, despite previous literature suggesting physical recovery may take up to 12 months [51], and high levels of caregiver burden reported by caregivers at six months post-surgery [37].There is currently little data regarding caregiver perspectives on the long-term recovery/care of hip fracture (for example, deconditioning and confidence in performing activities), thus understanding perspectives of the different caregiving trajectory, as people become more experienced carers and as people recover from hip fracture, would be valuable. Furthermore, it is unknown how long caregivers perceive the responsibility of caring for a person with hip fracture lasts for. No participant expressed an awareness of caring for an acute period of time and therefore how long this might last [52]. We acknowledge findings may not be fully generalisable because of the small sample size and England-based focus. Participants presented with limited socioeconomic, cultural or ethic perspectives, despite our best endeavours and there was also a lack of perspectives of those caring for a person with cognitive impairment [53]. This is important as such differences may be a source of variation in attitudes, behaviours, and perspectives of caregiving for someone following hip fracture [54,55]. We also appreciate that the present cohort was largely of lower mean age than may be expected for a hip fracture population, however this work provides more novel insights into the experiences of caregiving for younger people which is a profile anecdotally noted in practice [56]. Interviews were undertaken with informal caregivers and people with hip fracture together. This has limitations in that some individuals may be reluctant to address difficulties in the presence of the other person, some may assert dominance or reflect a particular status, and there is a likelihood of dyad interviews to be 'off-topic', compared to one-to-one interviews [57].

CONCLUSIONS

Whilst there may be an expectation by informal caregivers will provide support for people following hip fracture, our study illuminates challenges faced due to the acute and traumatic nature of hip fracture. We suggest that joint decision-making and goal setting with health professionals, patients and their informal carers will enable better preparedness for the transition from hospital to home. This includes a greater explanation and tailoring of the recovery pathway especially for younger hip fracture patients.

For peer review only - http://bmjopen.bmj.com/site/about/guidelines.xhtml

ACKNOWLEDGEMENTS AND DECLARATIONS

Collaborators: The HIP HELPER Study Collaborators: Penny Clifford (Norfolk, PPI Representative), Lis Freeman (Norfolk, PPI Representative), Rene Gray (Principal Investigator – James Paget University Hospital NHS Trust), Yan Cunningham (Principal Investigator – City Hospitals Sunderland NHS Foundation Trust), Sarah Langford (Principal Investigator - Northumbria Healthcare NHS Foundation Trust), Dr Mark Baxter (Principal Investigator - University Hospital Southampton NHS Foundation Trust), Jessica Pawson – (Principal Investigator - Barts Health NHS Trust), Melissa Taylor (James Paget University Hospital NHS Trust), Anna Mellows (James Paget University Hospital NHS Trust), Kate Lacey (James Paget University Hospital NHS Trust), Alex Herring (City Hospital Sunderland NHS Foundation Trust), Diane Williams (Northumbria Healthcare NHS Foundation Trust), Anna Cromie (Northumbria Healthcare NHS Foundation Trust), Gail Menton (Northumbria Healthcare NHS Foundation Trust), Warren Corbett (University Hospital Southampton NHS Foundation Trust), Helen Jowett (University Hospital Southampton NHS Foundation Trust), Vishwanath Joshi (Barts Health NHS Trust), Maninderpal Matharu (Barts Health NHS Trust), Maria Baggot (University Hospital Southampton NHS Foundation Trust) and David Barker (University Hospital Southampton NHS Foundation Trust).

Oversight Committee Membership: TOC Members: Associate Professor Susan Dutton (Chair; University of Oxford, UK), Professor Opinder Sahota (University of Nottingham, UK), Dr Katie Sheehan (Kings College London, UK).

Authors' Contributors: SHa, AW, TS, SHo, KP, RK, PA, PL, MCo, SEL, MCr researched the topic and devised the study. TS, SHa, RK, PA, AC, SHo, AW, KP, PL, MCo, SEL, MCr provided the first draft of the manuscript. SHa lead the qualitative study design. AW lead the qualitative research conduct. TS, SHa, AW, RK, PA, AC, SHo, KP, PL, MCo, SEL, MCr contributed equally to manuscript preparation. TS acts a guarantor.

Funding Statement: This project is funded by the National Institute for Health and Care Research (NIHR) under its Research for Patient Benefit (RfPB) Programme (Grant Reference Number NIHR200731). MCo and SHo role in this study was supported by the National Institute for Health and Care Research Oxford Biomedical Research Centre. SEL role in this study was supported by the National Institute for Health and Care Research Exeter Biomedical Research Centre. The views expressed are those of the author(s) and not necessarily those of the NIHR or the Department of Health and Social Care.

Competing Interests Statement: None declared.

Disclaimer: MCo and SHo are supported by the National Institute for Health Research (NIHR) Oxford Biomedical Research Centre (BRC). SEL is supported by the National Institute for Health and Care Research Exeter Biomedical Research Centre. The views expressed are those of the author(s) and not necessarily those of the NHS, the NIHR or the Department of Health and Social Care.

Ethics Approval: North East - Newcastle & North Tyneside 1 Research Ethics Committee (20/NE/0213) Date: 16 March 2021. Written informed consent was obtained from all participants.

Trial Sponsor: University of East Anglia (Research Sponsor, Research and Innovation Services, The Registry, University of East Anglia, Norwich, NR4 7TJ; Email: researchsponsor@uea.ac.uk) The views expressed are those of the author(s) and not necessarily those of the sponsor.

Provenance and Peer Review: Not commissioned; externally peer reviewed.

For peer review only - http://bmjopen.bmj.com/site/about/guidelines.xhtml

Data availability statement: The data that support the findings of this study are available from the corresponding author (AW) upon reasonable request. This includes access to the full protocol, and anonymised participant-level dataset.

Patient consent for publication: Not required.

For peer review only

REFERENCES

1 Lee H, Cook JA, Lamb SE, *et al.* The findings of a surgical hip fracture trial were generalizable to the UK national hip fracture database. *J Clin Epidemiol* 2021;**131**. doi:10.1016/j.jclinepi.2020.11.016

2 Baker PN, Salar O, Ollivere BJ, *et al.* Evolution of the hip fracture population: Time to consider the future? A retrospective observational analysis. *BMJ Open* 2014;**4**. doi:10.1136/bmjopen-2013-004405

3 Lawler K, Taylor NF, Shields N. Involving family members in physiotherapy for older people transitioning from hospital to the community: A qualitative analysis. *Disabil Rehabil* 2015;**37**. doi:10.3109/09638288.2014.996673

4 Griffin XL, Parsons N, Achten J, *et al.* Recovery of health-related quality of life in a United Kingdom hip fracture population: The warwick hip trauma evaluation - A prospective cohort study. *Bone and Joint Journal* 2015;**97-B**. doi:10.1302/0301-620X.97B3.35738

5 Seitz DP, Adunuri N, Gill SS, *et al.* Prevalence of Dementia and Cognitive Impairment Among Older Adults With Hip Fractures. J Am Med Dir Assoc. 2011;**12**. doi:10.1016/j.jamda.2010.12.001

6 Em S, Bozkurt M, Caglayan M, *et al.* Psychological health of caregivers and association with functional status of stroke patients. *Top Stroke Rehabil* 2017;**24**. doi:10.1080/10749357.2017.1280901

7 Stajduhar KI, Funk L, Toye C, *et al.* Part 1: Home-based family caregiving at the end of life: A comprehensive review of published quantitative research (1998-2008). Palliat Med. 2010;**24**. doi:10.1177/0269216310371412

8 Erickson ME. Effects of motivation, roles, coping strategies, and adaptations in relationships and personality on caretaking of elderly parents by midlife couples. Dissertation Abstracts International Section A: Humanities and Social Sciences. 2002;**62**.

9 Albertini M, Kohli M, Vogel C. Intergenerational transfers of time and money in European families: Common patterns - Different regimes? *J Eur Soc Policy* 2007;**17**. doi:10.1177/0958928707081068

10 Gérain P, Zech E. Informal Caregiver burnout? Development of a theoretical framework to understand the impact of caregiving. *Front Psychol* 2019;**10**. doi:10.3389/fpsyg.2019.01748

11 Sutter-Leve R, Passint E, Ness D, *et al.* The caregiver experience after stroke in a covid-19 environment: A qualitative study in inpatient rehabilitation. *Journal of Neurologic Physical Therapy* 2021;**45**. doi:10.1097/NPT.0000000000000336

12 Feast A, Moniz-Cook E, Stoner C, *et al.* A systematic review of the relationship between behavioral and psychological symptoms (BPSD) and caregiver well-being. Int Psychogeriatr. 2016;**28**. doi:10.1017/S1041610216000922

13 Saletti-Cuesta L, Tutton E, Langstaff D, *et al.* Understanding informal carers' experiences of caring for older people with a hip fracture: a systematic review of qualitative studies. *Disabil Rehabil* 2018;**40**:740–50. doi:10.1080/09638288.2016.1262467

14 Hawkins RJ, Jowett A, Godfrey M, *et al.* Poststroke Trajectories: The Process of Recovery Over the Longer Term Following Stroke. *Glob Qual Nurs Res* 2017;**4**:233339361773020. doi:10.1177/2333393617730209

15 Sheehan KJ, Smith TO, Martin FC, *et al.* Conceptual Framework for an Episode of Rehabilitative Care After Surgical Repair of Hip Fracture. *Phys Ther* 2019;**99**:276–85. doi:10.1093/ptj/pzy145

16 Smith T, Clark L, Khoury R, *et al.* A feasibility study to assess the design of a multicentre randomized controlled trial of the clinical and cost-effectiveness of a caregiving intervention for people following hip fracture surgery. *Bone Jt Open* 2021;**2**. doi:10.1302/2633-1462.211.BJO-2021-0136

For peer review only

For peer review only - http://bmjopen.bmj.com/site/about/guidelines.xhtml

17 Tong A, Sainsbury P, Craig J. Consolidated criteria for reporting qualitative research (COREQ): A 32-item checklist for interviews and focus groups. *International Journal for Quality in Health Care* 2007;**19**. doi:10.1093/intqhc/mzm042

18 Ariza-Vega P, Ortiz-Piña M, Kristensen MT, *et al.* High perceived caregiver burden for relatives of patients following hip fracture surgery. *Disabil Rehabil* 2019;**41**. doi:10.1080/09638288.2017.1390612

19 Wimo A, Wetterholm AL, Mastey V, *et al.* Evaluation of the resource utilization and caregiver time in Anti-dementia drug trials—a quantitative battery. In: *The Health Economics of Dementia*. 1998.

20 Braun V, Clarke V. One size fits all? What counts as quality practice in (reflexive) thematic analysis? *Qual Res Psychol* 2021;**18**:328–52. doi:10.1080/14780887.2020.1769238

21 Charmaz C. *Constructing Grounded Theory*. 2nd ed. 2014.

22 Hanson S, Gilbert D, Landy R, *et al.* Cancer risk in socially marginalised women: An exploratory study. *Soc Sci Med* 2019;**220**:150–8. doi:10.1016/j.socscimed.2018.11.009

23 Skivington K, Matthews L, Simpson SA, *et al.* A new framework for developing and evaluating complex interventions: Update of Medical Research Council guidance. *The BMJ* 2021;**374**. doi:10.1136/bmj.n2061

24 Braun V, Clarke V. To saturate or not to saturate? Questioning data saturation as a useful concept for thematic analysis and sample-size rationales. *Qual Res Sport Exerc Health* 2021;**13**:201–16. doi:10.1080/2159676X.2019.1704846

25 Mauthner NS, Doucet A. Reflexive Accounts and Accounts of Reflexivity in Qualitative Data Analysis. *Sociology* 2003;**37**:413–31. doi:10.1177/00380385030373002

26 Hagens V, Dobrow MJ, Chafe R. Interviewee Transcript Review: assessing the impact on qualitative research. *BMC Med Res Methodol* 2009;**9**:47. doi:10.1186/1471-2288-9-47

27 Thomas DR. Feedback from research participants: are member checks useful in qualitative research? *Qual Res Psychol* 2017;**14**:23–41. doi:10.1080/14780887.2016.1219435

28 Tutton E, Saletti-Cuesta L, Langstaff D, *et al.* Patient and informal carer experience of hip fracture: a qualitative study using interviews and observation in acute orthopaedic trauma. *BMJ Open* 2021;**11**:e042040. doi:10.1136/bmjopen-2020-042040

29 Zarzycki M, Seddon D, Bei E, *et al.* Why do they care? A qualitative systematic review and meta-synthesis of personal and relational motivations for providing informal care. *Health Psychol Rev* 2022;:1–33. doi:10.1080/17437199.2022.2058581

30 Cobley CS, Fisher RJ, Chouliara N, *et al.* A qualitative study exploring patients' and carers' experiences of Early Supported Discharge services after stroke. *Clin Rehabil* 2013;**27**. doi:10.1177/0269215512474030

31 Isik AT, Soysal P, Solmi M, *et al.* Bidirectional relationship between caregiver burden and neuropsychiatric symptoms in patients with Alzheimer's disease: A narrative review. In: *International Journal of Geriatric Psychiatry*. 2019. doi:10.1002/gps.4965

32 Kuzuya M, Enoki H, Hasegawa J, *et al.* Impact of caregiver Burden on adverse health outcomes in community-dwelling dependent older care recipients. *American Journal of Geriatric Psychiatry* 2011;**19**. doi:10.1097/JGP.0b013e3181e9b98d

33 Naylor MD, Shaid EC, Carpenter D, *et al.* Components of Comprehensive and Effective Transitional Care. *J Am Geriatr Soc* 2017;**65**. doi:10.1111/jgs.14782

34 Guilcher SJT, Everall AC, Cadel L, *et al.* A qualitative study exploring the lived experiences of deconditioning in hospital in Ontario, Canada. *BMC Geriatr* 2021;**21**. doi:10.1186/s12877-021-02111-2

35 Angeli JM, Harpster KL, Hanson E, *et al.* Patient- and caregiver-identified preferences: Dimensions of change in developmental therapy treatment goals. *Dev Neurorehabil* 2019;**22**. doi:10.1080/17518423.2018.1425754

For peer review only - http://bmjopen.bmj.com/site/about/guidelines.xhtml

36 Brewer K, Pollock N, Wright FV. Addressing the challenges of collaborative goal setting with children and their families. *Phys Occup Ther Pediatr* 2014;**34**. doi:10.3109/01942638.2013.794187

37 Parry JA, Langford JR, Koval KJ. Caregivers of hip fracture patients: The forgotten victims? *Injury* 2019;**50**. doi:10.1016/j.injury.2019.09.030

38 Ding TYG, De Roza JG, Chan CY, *et al.* Factors associated with family caregiver burden among frail older persons with multimorbidity. *BMC Geriatr* 2022;**22**. doi:10.1186/s12877-022-02858-2

39 Yuen EYN, Knight T, Ricciardelli LA, *et al.* Health literacy of caregivers of adult care recipients: A systematic scoping review. Health Soc Care Community. 2018;**26**. doi:10.1111/hsc.12368

40 Kim Y. Korean-American family postcaregivers on dementia caregiving: A phenomenological inquiry. *J Gerontol Soc Work* 2009;**52**. doi:10.1080/01634370903048352

41 Kuşçu MK, Dural U, Yaşa Y, *et al.* Decision pathways and individual motives in informal caregiving during cancer treatment in Turkey. *Eur J Cancer Care (Engl)* 2009;**18**. doi:10.1111/j.1365-2354.2007.00900.x

42 Øydgard GW. The influence of institutional discourses on the work of informal carers: An institutional ethnography from the perspective of informal carers. *BMC Health Serv Res* 2017;**17**. doi:10.1186/s12913-017-2591-7

43 Stajduhar KI, Martin WL, Barwich D, *et al.* Factors influencing family caregivers' ability to cope with providing end-of-life cancer care at home. *Cancer Nurs* 2008;**31**. doi:10.1097/01.NCC.0000305686.36637.b5

44 Zarzycki M, Morrison V, Bei E, *et al.* Cultural and societal motivations for being informal caregivers: a qualitative systematic review and meta-synthesis. *Health Psychol Rev* Published Online First: 2022. doi:10.1080/17437199.2022.2032259

45 Bueno M, Chase J-A. Gender Differences in Caregiving of Older Adults: A Systematic Review of the Literature. *Innov Aging* 2020;**4**. doi:10.1093/geroni/igaa057.491

46 Boonsin S, Deenan A, Wacharasin C. Factors influencing the burden of family caregiving for survivors of stroke. *Pac Rim Int J Nurs Res Thail* 2020;**25**.

47 Le DD, Ibuka Y. Understanding the effects of informal caregiving on health and well-being: Heterogeneity and mechanisms. *Soc Sci Med* 2023;**317**:115630. doi:10.1016/j.socscimed.2022.115630

48 Alpantaki K, Papadaki C, Raptis K, *et al.* Gender and Age Differences in Hip Fracture Types among Elderly: a Retrospective Cohort Study. *Maedica (Bucur)* 2020;**15**. doi:10.26574/maedica.2020.15.2.185

49 Fox F, Drew S, Gregson C, *et al.* Complex organisational factors influence multidisciplinary care for patients with hip fractures: a qualitative study of barriers and facilitators to service delivery. *BMC Musculoskelet Disord* 2023;**24**:128. doi:10.1186/s12891-023-06164-9

50 Griffiths F, Mason V, Boardman F, *et al.* Evaluating recovery following hip fracture: a qualitative interview study of what is important to patients. *BMJ Open* 2015;**5**:e005406–e005406. doi:10.1136/bmjopen-2014-005406

51 Magaziner J, Chiles N, Orwig D. Recovery after Hip Fracture: Interventions and Their Timing to Address Deficits and Desired Outcomes-Evidence from the Baltimore Hip Studies. *Nestle Nutr Inst Workshop Ser* 2015;**83**. doi:10.1159/000382064

52 Cameron JI, Gignac MAM. "Timing It Right": A conceptual framework for addressing the support needs of family caregivers to stroke survivors from the hospital to the home. *Patient Educ Couns* 2008;**70**:305–14. doi:10.1016/j.pec.2007.10.020

53 Francis N, Hanna P. Informal carer experiences of UK dementia services—A systematic review. J Psychiatr Ment Health Nurs. 2022;**29**. doi:10.1111/jpm.12698

54 Fernández-Ballesteros R, Sánchez-Izquierdo M, Olmos R, *et al.* Cultural stereotypes in care contexts. *Clin Interv Aging* 2018;**13**. doi:10.2147/CIA.S169487

For peer review only - http://bmjopen.bmj.com/site/about/guidelines.xhtml

For peer review only

55 Dilworth-Anderson P, Williams IC, Gibson BE. Issues of race, ethnicity, and culture in caregiving research: A 20-year review (1980-2000). Gerontologist. 2002;**42**. doi:10.1093/geront/42.2.237

56 Veronese N, Maggi S. Epidemiology and social costs of hip fracture. *Injury* 2018;**49**. doi:10.1016/j.injury.2018.04.015

57 Szulc J, King N. The Practice of Dyadic Interviewing: Strengths, Limitations and Key Decisions. *Forum Qualitative Sozialforschung Forum: Qualitative Social Research* 2022;**23**.

For peer review only

Table 1: Characteristics of sample interviewed

	Intervention Group N=7	Control Group N=3	Overall N=10
**Informal Caregiver**			
Age (years): median (IQR)	71.0 (58.0, 81.0)	71.0 (43.0, 72.0)	71.0 (58.0, 77.0)
Gender: n (%)			
- Male	5 (71.4%)	2 (66.7%)	7 (70.0%)
- Female	2 (28.6%)	1 (33.3%)	3 (30.0%)
Ethnicity: n (%)			
- White British	6 (85.7%)	3 (100%)	9 (90.0%)
- White other	1 (14.3%)	0	1 (10.0%)
Relationship to person with hip fracture: n (%)	4 (57.1%)	2 (66.7%)	6 (60.0%)
- Spouse	1 (14.3%)	1 (33.3%)	2 (20.0%)
- Daughter/son	2 (28.6%)	0	2 (20.0%)
- Other			
Employment: n (%)			
- Not working	6 (85.7%)	3 (100%)	9 (90.0%)
- Part-time work	1 (14.3%)	0	1 (10.0%)
**Person with Hip Fracture**			
Age at consent (years): median (IQR)	79.0 (70.0, 82.0)	69.0 (68.0, 71.0)	72.5 (69.0, 79.0)
Gender: n (%)			
- Male	1 (14.3%)	0	1 (10.0%)
- Female	6 (85.7%)	3 (100%)	9 (90.0%)
Ethnicity: n (%)			
- White British	7 (100%)	3 (100%)	10 (100%)
Has Cognitive Impairment (based on AMTS category): n (%)	1 (14.3%)	0	1 (10.0%)
AMTS score at consent: median (IQR)	10.0 (9.0, 10.0)	10.0 (10.0, 10.0)	10.0 (9.0, 10.0)
NEADL score at baseline: median (IQR)	20.0 (14.0, 22.0)	10.0 (10.0, 10.0)	17.0 (12.0, 22.0)

**Location**		
Site (n)		
1	1	1
2	1	1
3	1	1
4	3	-
5	1	-

AMTS – Abbreviated Mental Test Score; NEADL – Nottingham Extended Activities of Daily Living scale

For peer review only

For peer review only - http://bmjopen.bmj.com/site/about/guidelines.xhtml

Topic	Sample questions	Prompts/probes
Overall	• Overall, do share your experiences of being involved with our research?	
The approach and consent process	• Please talk me through how you got involved with the HipHelper research study?<br>• How clearly was the study explained to you?	• Could we have dealt with that differently?<br>• How was this different to what you expected?<br>• For the future, what could we improve about the description of the study?
Willingness to be randomised to either group/ participant views of their intervention<br><br>The risk of intervention contamination between the groups	• Please talk me through what study treatment you received<br><br>• How much did talk to any other patients or caregivers (whilst in hospital) about the intervention?	• *If required: clarify what HIP HELPER was and what was usual care/non-study intervention*<br>• You were allocated to X group. What did that feel like?<br>• What discussions were had between those who received it and did not receive it?
**Both groups**		
The acceptability of the inpatient care	• Please talk me through your treatment while you were in the hospital	• What aspects of your care that were lacking in the hospital?<br>• Can you remember which wards you were on? Can you remember being moved to different areas of the hospital?

For peer review only

Topic	Sample questions	Prompts/probes
	• *(Carers)* As X's carer, what was your impression of the care?	• For both of you, what was helpful and less helpful to your care?
**Intervention group only**		
In-patient HIP HELPER programme<br><br>Strengths/weaknesses	• How did you find the HIP HELPER programme?<br><br>• Please tell me what you remember about the manual handling training you had in the hospital?<br>• Please tell me about the phone calls you received?<br><br>• What were the individualised goals you set in the hospital?	• Please share any specific examples of what you can remember from the programme?<br>• What were the most helpful bits of your HIP HELPER intervention?<br>• What was good about it?<br>• What were the less helpful/worse bits of the HIP HELPER intervention?<br>• For you as the patient/and you as X's carer?<br>• How did you get on with that at home?<br>• Was it helpful? If so, how?<br><br>• Can you remember what you talked about?<br>• Can you give specific examples of what was helpful?<br>• Was there any advice that confused you or you weren't clear about?<br>• How did you come up with these?<br>• *(Carer)* By how far were you involved in the goal setting process?<br>• In retrospect, what goal(s) would you alter?

For peer review only

Topic	Sample questions	Prompts/probes
**Both groups:**		
On return to your home	• *(If required, state residential status)* Can you tell me who you live with at home?	
	• What were the first things you wanted to achieve once at home?<br>• How did you decide who was to be your allocated carer?<br>• *(Carers)* Can you talk me through this decision to be designated as X's carer?	• On a 1-10 scale, how confident did you feel when you left the hospital?<br>• Can you tell me why you have chosen that number on the scale?
	• *(Long-term carers)* Once at home, how do you feel care-giving role changed?	• Can you tell me about any new caring responsibilities?<br>• How did you organise these?<br>• Did this change the dynamics in their relationship in taking on caring role, if so, how
	• What sort of adaptions did you make at home to enable you to move about independently (where possible)?<br>• What do you think is lacking in the transition from hospital to home?	• Are/were there any other things that may have affected your transfer home?<br>• Are/were there any other things that required you to adapt the advice you were given?
	• How willing were/are you to continue with activities/advice that were suggested to you?<br>• Once at home, by how far did you feel supported by the health care professions you had seen?	*Probes: transferring, dressing, activities of daily living*

For peer review only

Topic	Sample questions	Prompts/probes
	• *(Carers)* For you as caregiver, how did you get on with helping move X about?<br>• Were there any adaptions that you made to the advice given?	
What modifications they may recommend to interventions received	• What changes did you find easy to implement and what did you find harder?	• What could we improve?
	• What do we need to modify for any future programmes to support people with a hip replacement and their carer to help you get up and moving and returning to normal life?	• Can you explain why? *Prompt around ADL's*
The ease and convenience of the data collection processes/applicability of the methods and measures used	• As you were part of a trial, we had to collect a lot of measurements. Can you talk me through what these were?	*Prompt:*<br>*'Share screen' of the front page of questionnaires to help with memory recall.*
	• How did you manage with the questionnaires we gave you at the start of the study and at the end in the post?<br>• What about your interview experience today?	• Were they easy to complete?<br>• Do you remember them being a problem?<br>• How convenient were they?<br>• How did you find it?<br>• Is there anything we can do to improve this experience?
Summary and end	• We are now ? months since your hip replacement, by how far do you think the HipHelper programme (*or usual care)* has helped with your recovery?<br>• If we were to run this programme again in the future, is there anything else we should consider?	• How far do you think the Hip Helper programme (*or usual care)* has helped you achieve your goals?

For peer review only

Topic	Sample questions	Prompts/probes
	• How do you think we could better support you and your carer to recover after hip surgery?<br>• Is anything else you have gained by being involved in this research study<br>• Is there anything else we haven't thought of and that you can share with us to improve the study and the care of hip replacement patients and their carers?	

For peer review only

COREQ (COnsolidated criteria for REporting Qualitative research) Checklist

A checklist of items that should be included in reports of qualitative research. You must report the page number in your manuscript where you consider each of the items listed in this checklist. If you have not included this information, either revise your manuscript accordingly before submitting or note N/A.

Topic	Item No.	Guide Questions/Description	Reported on Page No.
**Domain 1: Research team and reflexivity**			
Personal characteristics			
Interviewer/facilitator	1	Which author/s conducted the interview or focus group?	
Credentials	2	What were the researcher's credentials? E.g. PhD, MD	
Occupation	3	What was their occupation at the time of the study?	
Gender	4	Was the researcher male or female?	
Experience and training	5	What experience or training did the researcher have?	
Relationship with participants			
Relationship established	6	Was a relationship established prior to study commencement?	
Participant knowledge of the interviewer	7	What did the participants know about the researcher? e.g. personal goals, reasons for doing the research	
Interviewer characteristics	8	What characteristics were reported about the inter viewer/facilitator? e.g. Bias, assumptions, reasons and interests in the research topic	
**Domain 2: Study design**			
Theoretical framework			
Methodological orientation and Theory	9	What methodological orientation was stated to underpin the study? e.g. grounded theory, discourse analysis, ethnography, phenomenology, content analysis	
Participant selection			
Sampling	10	How were participants selected? e.g. purposive, convenience, consecutive, snowball	
Method of approach	11	How were participants approached? e.g. face-to-face, telephone, mail, email	
Sample size	12	How many participants were in the study?	
Non-participation	13	How many people refused to participate or dropped out? Reasons?	
Setting			
Setting of data collection	14	Where was the data collected? e.g. home, clinic, workplace	
Presence of non-participants	15	Was anyone else present besides the participants and researchers?	
Description of sample	16	What are the important characteristics of the sample? e.g. demographic data, date	
Data collection			
Interview guide	17	Were questions, prompts, guides provided by the authors? Was it pilot tested?	
Repeat interviews	18	Were repeat inter views carried out? If yes, how many?	
Audio/visual recording	19	Did the research use audio or visual recording to collect the data?	
Field notes	20	Were field notes made during and/or after the inter view or focus group?	
Duration	21	What was the duration of the inter views or focus group?	
Data saturation	22	Was data saturation discussed?	
Transcripts returned	23	Were transcripts returned to participants for comment and/or	

For peer review only

Topic	Item No.	Guide Questions/Description	Reported on Page No.
		correction?	
**Domain 3: analysis and findings**			
Data analysis			
Number of data coders	24	How many data coders coded the data?	
Description of the coding tree	25	Did authors provide a description of the coding tree?	
Derivation of themes	26	Were themes identified in advance or derived from the data?	
Software	27	What software, if applicable, was used to manage the data?	
Participant checking	28	Did participants provide feedback on the findings?	
Reporting			
Quotations presented	29	Were participant quotations presented to illustrate the themes/findings? Was each quotation identified? e.g. participant number	
Data and findings consistent	30	Was there consistency between the data presented and the findings?	
Clarity of major themes	31	Were major themes clearly presented in the findings?	
Clarity of minor themes	32	Is there a description of diverse cases or discussion of minor themes?	

Developed from: Tong A, Sainsbury P, Craig J. Consolidated criteria for reporting qualitative research (COREQ): a 32-item checklist for interviews and focus groups. *International Journal for Quality in Health Care*. 2007. Volume 19, Number 6: pp. 349 – 357

Once you have completed this checklist, please save a copy and upload it as part of your submission. DO NOT include this checklist as part of the main manuscript document. It must be uploaded as a separate file.

For peer review only

BMJ Open

Perspectives of informal caregivers who support people following hip fracture surgery: a qualitative study embedded within the HIP HELPER feasibility trial

Journal:	*BMJ Open*
Manuscript ID	bmjopen-2023-074095.R2
Article Type:	Original research
Date Submitted by the Author:	27-Sep-2023
Complete List of Authors:	Welsh, Allie; University of East Anglia, School of Education<br>Hanson, Sarah; University of East Anglia, School of Health Sciences<br>Pfeiffer, K; Robert Bosch Hospital, Department of Clinical Gerontology and Geriatric Rehabilitation<br>Khoury, Reema; University of East Anglia,<br>Clark, Allan; University of East Anglia, Norwich Medical School<br>Ashford, Polly-Anna; University of East Anglia, Norwich Clinical Trials Unit<br>Hopewell, Sally; University of Oxford, Nuffield Department of Orthopaedics, Rheumatology and Musculoskeletal Sciences<br>Logan, Phillipa; University of Nottingham, Community Health Sciences<br>Crotty, Maria; Flinders University, Rehabilitation, Aged and Extended Care<br>Costa, Matthew; University of Oxford, Nuffield Department of Orthopaedics, Rheumatology, and Musculoskeletal Sciences<br>Lamb, Sarah; University of Exeter, College of Medicine and Health<br>Smith, Toby; University of Warwick; University of East Anglia, School of Health Sciences<br>Collaborators, HIPHELPER ; University of East Anglia, School of Health Sciences
**Primary Subject Heading**:	Surgery
Secondary Subject Heading:	Qualitative research, Rehabilitation medicine
Keywords:	Hip < ORTHOPAEDIC & TRAUMA SURGERY, QUALITATIVE RESEARCH, REHABILITATION MEDICINE

SCHOLARONE™
Manuscripts

I, the Submitting Author has the right to grant and does grant on behalf of all authors of the Work (as defined in the below author licence), an exclusive licence and/or a non-exclusive licence for contributions from authors who are: i) UK Crown employees; ii) where BMJ has agreed a CC-BY licence shall apply, and/or iii) in accordance with the terms applicable for US Federal Government officers or employees acting as part of their official duties; on a worldwide, perpetual, irrevocable, royalty-free basis to BMJ Publishing Group Ltd ("BMJ") its licensees and where the relevant Journal is co-owned by BMJ to the co-owners of the Journal, to publish the Work in this journal and any other BMJ products and to exploit all rights, as set out in our licence.

The Submitting Author accepts and understands that any supply made under these terms is made by BMJ to the Submitting Author unless you are acting as an employee on behalf of your employer or a postgraduate student of an affiliated institution which is paying any applicable article publishing charge ("APC") for Open Access articles. Where the Submitting Author wishes to make the Work available on an Open Access basis (and intends to pay the relevant APC), the terms of reuse of such Open Access shall be governed by a Creative Commons licence – details of these licences and which Creative Commons licence will apply to this Work are set out in our licence referred to above.

Other than as permitted in any relevant BMJ Author's Self Archiving Policies, I confirm this Work has not been accepted for publication elsewhere, is not being considered for publication elsewhere and does not duplicate material already published. I confirm all authors consent to publication of this Work and authorise the granting of this licence.

For peer review only

Perspectives of informal caregivers who support people following hip fracture surgery: a qualitative study embedded within the HIP HELPER feasibility trial

Authors: Welsh A,[1] Hanson S,[2] Pfeiffer K,[3] Khoury R,[2] Clark A,[2] Ashford P-A,[2] Hopewell S,[4] Logan PA,[5] Crotty M,[6] Costa ML,[4] Lamb SE,[7] Smith TO[2,8] on behalf of the HIP HELPER Study Collaborators.

Affiliations

1. School of Education, University of East Anglia, Norwich, UK
2. Faculty of Medicine and Health Sciences, University of East Anglia, Norwich, UK
3. Department of Geriatric Rehabilitation, Robert-Bosch-Hospital, Stuttgart, Germany
4. Nuffield Department of Rheumatology, Orthopaedics and Musculoskeletal Sciences, University of Oxford, Oxford, UK
5. School of Medicine, University of Nottingham, Nottingham, UK
6. College of Medicine and Public Health, Flinders University, Adelaide, Australia
7. College of Medicine and Health Sciences, University of Exeter, Exeter, UK
8. Warwick Medical School, University of Warwick, Coventry, UK

Correspondence to:
Dr Allie Welsh, School of Education, University of East Anglia, Norwich, NR4 7TJ, UK
Email: a.welsh@uea.ac.uk

Word counts: Manuscript: 4335; Abstract: 252

For peer review only - http://bmjopen.bmj.com/site/about/guidelines.xhtml

ABSTRACT

Objectives: To illuminate the perspectives of informal caregivers who support people following hip fracture surgery.

Design: A qualitative study embedded within a now completed multi-centre, feasibility randomised controlled trial (HIP HELPER).

Setting: Five English National Health Service (NHS) hospitals.

Participants: We interviewed 20 participants (10 informal caregivers; 10 people with hip fracture), following hip fracture surgery. This included one male and nine females who experienced a hip fracture; and seven male and three female informal caregivers. The median age was 72.5 years (range: 65 to 96 years), 71.0 years (range: 43 to 81 years) for people with hip fracture and informal caregivers, respectively.

Methods: Semi-structured, virtual interviews were undertaken between November 2021 and March 2022, with caregiver dyads (person with hip fracture and their informal caregiver). Data were analysed thematically.

Findings: We identified two main themes: expectations of the informal caregiver role, and reality of being an informal caregiver; and sub-themes: expectations of care and services; responsibility and advocacy; profile of people with hip fracture; decision to be a caregiver; transition from hospital to home.

Conclusion: Findings suggest informal caregivers do not feel empowered to advocate for a person's recovery or navigate the care system, leading to increased and unnecessary stress, anxiety and frustration when supporting the person with hip fracture. We suggest that a tailored information-giving on the recovery pathway, which is responsive to the caregiving population (i.e., considering the needs of male, younger, and more active informal caregivers and people with hip fracture) would smooth the transition from hospital to home.

Trial registration number: ISRCTN13270387 (HIP HELPER).

Keywords: informal caregivers; hip fracture; recovery; rehabilitation; carer; qualitative

For peer review only

STRENGTHS AND LIMITATIONS OF THIS STUDY

- We describe the contextual factors of participating in HIP HELPER and people's experiences of giving and receiving informal care.
- This study may help health professionals support informal caregiver dyads in the transition from hospital to home.
- Participants presented with limited socioeconomic, cultural or ethic perspectives, and there was a lack of perspectives of those caring for a person with cognitive impairment.
- The COVID-19 pandemic affected NHS services which may have impacted on the study delivery and participant's experiences.

INTRODUCTION

Hip fracture is a devastating injury, predominantly seen in frail older people, and disproportionally more women [1]. Approximately 80,000 people aged 60 years and over experience a hip fracture in the UK each year, resulting in a combined health and social cost of at least £2 billion [2]. This cost is expected to increase with an ageing population [1]. Outcomes following hip fracture are poor. The majority of individuals do not return to pre-injury levels of function, and frequently lose independence and self-caring abilities [3,4].

Given the sudden, traumatic nature of hip fracture, people often rely on family members or friends for informal care following hospital discharge. Additionally, approximately 40% of people who sustain a hip fracture have cognitive impairment, meaning many caregivers also have to cope with the challenges of functional dependency and neurocognitive symptoms [5]. These 'informal caregivers' therefore play an integral role, in the transition from hospital to home, the initial discharge period and possibly beyond [6].

Informal caregivers are a heterogeneous population. For some, this may be the first time with caregiving responsibilities; for others, it is perceived as an episodic event with a temporary increase in care needs. They may be a retired spouse, friend, grown-up children in employment; living with, close by or far away; and of course, they be facing their own physical and mental health challenges. Caregiving may further be exacerbated by (but not limited to) the ageing population, smaller average family size, changing gender-roles due in part to more women working outside the home [7–9].

The ability of a person to cope with the roles and responsibility of caregiving is a complex interaction of different factors. In addition to stressors related to the actual caregiving, there are other factors that may determine a person's ability to manage the caregiving role, such as: caregiver resources, social environment (e.g., informal and professional support), relationship quality with the care-recipient and caregiver, positive aspects of caregiving (e.g., finding meaning in the caregiving role) [10]. Whilst caregiving experiences have been reported in other populations such as stroke [6,11] and dementia [12], there is still much to understand about acute episodes of informal care, such as in hip fracture. Previous literature suggests that the experiences of recovery after hip fracture for patients and informal caregivers may be different to these previous populations [13]. These may include differing recovery trajectories and impairment [14], greater challenges with post-operative pain management, nausea and fear avoidance [15].

Our qualitative study investigated the informal caregiver perspectives of caring for a person with hip fracture following hospital discharge, nested within a larger study aimed at assessing the feasibility of

For peer review only - http://bmjopen.bmj.com/site/about/guidelines.xhtml

a pragmatic, multi-centre randomised controlled trial (RCT) of an informal caregiver training programme (HIP HELPER) to support the recovery of people following hip fracture surgery [16].

METHODS

This was a qualitative study embedded within a multi-centre, feasibility randomised controlled trial (HIP HELPER). The HIP HELPER protocol is published elsewhere, and the study is now complete [16]. In brief, HIP HELPER aimed to assess the design of a pragmatic RCT to test the effectiveness of an informal caregiver intervention, compared to usual NHS care following hip fracture surgery. Caregiver dyads who were randomised to receive the HIP HELPER intervention were allocated to received three, one- hour, one-to-one training sessions. Training session had standardised content and, all of which were delivered by a nurse, a physiotherapist, or an occupational therapist who had been trained to deliver the content. Training sessions included practical skills for rehabilitation such as: transfers and walking, pacing, and stress management techniques, and the provision of and the HIP HELPER Caregiver Workbook, offered information on recovery, exercises, worksheets, and goal-setting plans to facilitate a ‘good’ recovery.

The present study reports the experiences and contextual factors of undergoing hip fracture surgery and dyad’s experiences of giving and receiving informal care. Patient and caregiver perspective of the experimental caregiving training programme are to be reported with the feasibility study results. Group allocation (intervention or control) was therefore not a significant consideration for the present study. We followed the COREQ reporting guidelines [17].

Fourteen caregiver dyads were purposively sampled by age, pre-fracture disability, and hospital locations. Patients were aged 60 years and above, had undergone hip fracture surgery and had a nominated individual who acted as an informal caregiver. An informal caregiver was defined as someone who was expected to informally provide care, assistance, support or supervision in activities of daily living (ADLs) for at least three hours per week, but not on a paid basis. This may have included personal ADLs such as toileting, washing, dressing and eating; and/or more complex tasks such as managing money, shopping and household chores [18,19]. Other inclusion criteria for participants were community-dwelling prior to admission, able to attend face-to-face hospital appointments and/or access to a computer/table and internet services to receive a video consultation call.

Data collection

In-depth, semi-structured interviews were undertaken with caregiver dyads, within six-weeks of hospital discharge, by AW, who is a white, female post-doctoral researcher. AW had no role in recruitment to the study nor intervention delivery, thus was not known to participants. Interviews were conducted virtually using Microsoft Teams or via telephone, between November 2021 and March 2022. They were audio-recorded, transcribed and all identifying information removed.

It was not necessary to operationalise the concept of saturation, firstly given our sampling approach, and secondly given we recognise that meaning is generated through interpretation of data are inescapably situated and subjective [20,21]. As per Hanson et al’s [22] approach, we analysed the data to identify recurrent themes generated from the analysis. At 10 participant-dyads, we started to see recurring themes and ideas. Accordingly, we made a pragmatic decision that we had collected sufficient data to be representative of the hip fracture informal caregiver dyad experiences and ceased data collection.

Data analysis

For peer review only - http://bmjopen.bmj.com/site/about/guidelines.xhtml

Using the principles of thematic analysis, data were initially analysed deductively against our questioning framework and then further explored inductively to explore contextual features and participants experiences [23]. AW generated initial codes and themes. AW, SH, TS and KP then engaged in discussion to develop, review, refine and name themes.

Reflexivity was acknowledged in the design and analysis of our study. This included reflecting on the positions of researcher team (TS – physiotherapist, SHa – nurse, AW – physiologist, KP - psychologist) to ensure we understood the context and significance were interpreted appropriately and our predetermined positions were appreciated [24,25].

We did not return our transcripts to participants for comment or correction, and we did not seek ethical approval to do so. This is based on our experience as qualitative researchers and the little evidence available that suggests member checks improve research findings [26,27]. Participants were thus not involved in the data analysis process for these reasons and the lack of resources available for training individuals.

Patient and public involvement

Patient involvement began during protocol development and continued throughout the trial. The patient-member was a co-investigator, who provided insights into the trial conduct and supported the interpretation of findings during the trial's dissemination phase.

The topic guide was developed with two members of the public, who helped guide the approach and style of the interview topic guides through a pilot interview (**Supplementary File 1**). Learnings from the pilot included some rewording of questions for clarity and the use of prompts to further unpick experiences. As suggested by PPI members, the topic guide was shared with participants prior to the interview, enabling them to reflect and plan beforehand.

FINDINGS

Participants

In total, 10 interviews were undertaken with caregiver dyads (20 participants) across our five study sites in England. One dyad declined due to other commitments, one did not give a reason and the remaining dyads did not take part in the interview as the person with hip fracture had been readmitted to hospital. The median length of interviews was 33 minutes (range: 27 to 53 minutes).

Dyad characteristics are presented in **Table 1**. The median age of people with hip fracture and their caregiver was 72.5 years (range: 65 to 96 years) and 71.0 years (range: 43 to 81 years), respectively. Of the informal caregivers, seven were male and three were female. There were three males and seven females who experienced a hip fracture. Overall, six informal caregivers were the spouse of the person with hip fracture, two were adult children (a daughter and a son) and two were described as 'other'.

Themes

Overall, two main themes and associated sub-themes were identified:

(1) Expectations of the informal caregiver role

a) Expectations of care and services
b) Responsibility and advocacy

(2) Reality of being an informal caregiver
a) Profile of people with hip fracture
b) Decision to be a caregiver
c) Transition from hospital to home

Expectations of the informal caregiver role

Informal caregivers perceived that health professionals assumed that they would immediately take-on (additional) caregiving responsibilities upon the discharge home.

"Assumed that because I'm working from home [I would care], just said it like, didn't even ask me." (Caregiver, Female, Control Group)

a) Expectations of care and services

Both informal caregivers and people with hip fracture suggested that rather than being personalised, goals were set by health professionals on a 'standard' patient recovery trajectory and therefore these were not viewed as achievable, nor realistic. They also had insufficient information to understand what a typical recovery trajectory looks like. It appears that most people did not feel empowered and found goal setting and developing realistic recovery goals difficult, based on imperfect information.

"I had no expectations because I have never been in hospital before. Ever. I have never been ill before, so this is completely new to me and obviously new to [caregiver] – he's never had to care for me before." (Person with Hip Fracture, Female, Intervention Group)

"Not quite enough available to you about kind of next steps and what's going to happen in the future." (Caregiver, Male, Control Group)

In contrast, again for informal caregivers and people with hip fracture, receiving knowledge on recovery expectations with realistic goal-setting and joint decision-making, as seen in our HIP HELPER intervention, appeared to be empowering.

"If you get somebody who doesn't have sufficiently stimulating support, that is a problem because in a sense, it encourages them to stay dependent, whereas what it should be doing is encourage them to become independent." (Caregiver, Male, Intervention Group)

b) Responsibility and advocacy

Caregivers reported a sense that they must advocate and even battle for services to achieve adequate care and support in the transition from hospital to home, and yet, they had no power to do so. Navigating the care system is difficult and requires knowledge and confidence. This was particularly pertinent among first-time caregivers.

For peer review only - http://bmjopen.bmj.com/site/about/guidelines.xhtml

"There is only as much as I can do to protect my husband. He's got a right to have a minimum care. Really, we're not asking for much." (Caregiver, Female, Intervention Group)

"I had to make phone calls and chase around to try and sort things out [for her recovery at home]." (Caregiver, Female, Control Group)

Informal caregivers felt that this level of advocacy was time consuming and created an imbalance in undertaking care activities, thus detracting from giving personal support.

"But while I have to chase up all these things, he's not getting the best. He needs my attention, you know, to make sure that he's doing his exercise." (Caregiver, Female, Intervention Group)

Caregivers also expressed their feelings of accountability for supporting independence and recovery and, not surprisingly for some, this induced feelings of stress and anxiety.

"She's always been very independent, and I just felt very responsible for it. Seems to be quite a lot on my shoulders." (Caregiver, Male, Intervention Group)

"There was a shock when she fell and broke her hip. It's a lot to take on. All of a sudden, everything falls to one person, it's tough." (Caregiver, Male, Control Group)

<u>Reality of being an informal caregiver</u>

There was a dissonance in the expectations of being a caregiver and the actual role. The reality was shaped by the motivation and willingness to become a caregiver, the profile of the caregiver and the person with hip fracture, and the process of being discharged home. This mismatch was seen more in first-time caregivers and spouses of the person with hip fracture.

a) Profile of the caregiver and the person with hip fracture

The majority (n=8/10) of caregivers were first-time caregivers with little or no understanding of the hip fracture recovery process.

"His wife had Alzheimer's and he cared for her for about 16 years. So, it's something that he understands. And he, well, he's just very good at it." (Person with Hip Fracture, Female, Intervention Group)

For those caregivers in full-time employment, competing priorities between work and caring highlighted the reality of being an informal caregiver.

"I had loads of work on and I was having to work at 10 o'clock at night to try and get my work done, because through the day when places were open, I was fooling around trying to get the things we need, sort mam and do her appointments." (Caregiver, Female, Control Group)

The profile of people with hip fracture in this study, were generally active older adults, some of whom were still in employment and felt the services available post-hip fracture, were not conducive to their lifestyle, needs or goals. They perceived services are primarily designed for older adults, suggesting

For peer review only - http://bmjopen.bmj.com/site/about/guidelines.xhtml

inadequacy in rehabilitation for those with hip fracture (and their caregivers) aiming to return to occupational or physically demanding leisure activities.

*"I don't think we're typical patients and carers. Certainly, I am definitely not a typical carer, and *** is definitely not a typical patient."* (Caregiver, Male, Intervention Group)

"You know, I'm not a little old lady who sits and knits in the chair and doesn't move about much so that makes a difference, doesn't it?" (Person with Hip Fracture, Female, Intervention Group)

Caregivers were also mindful of their own age and health status. Caregivers were aware that they required physical and mental capacity to effectively care for the person following hip fracture and that maintaining their own health and wellbeing was equally as important.

"I was conscious that I had to take care of myself to be fit to be looking after her." (Caregiver, Male, Intervention Group)

"You know I'm so lucky that I'm healthy still. That I'm young and capable, but, who knows, you know?" (Caregiver, Male, Intervention Group)

Caregivers recognised the need to balance their own physical and psychological needs with their care-recipient's need.

b) Decision to be a caregiver

Findings identified multiple factors which contributed to the decision to become an informal caregiver. Spousal obligation and acting out of affection, were examples identified from the present study. For some, values such as loyalty and commitment arising from marriage (spousal obligation) was what motivated people to take on a caregiving.

"We both met two and a half years ago. Dare I say it nuts about each other? Ever since then, we've been living together, as man and wife." (Caregiver, Male, Intervention Group)

For others, there was the absence of *actual choice*, in-part attributed to assumptions made on discharge planning, but also underpinned by the unavailability of alternative care options.

"I think in our case there wouldn't have been anyone else who would be able to come and help." (Caregiver, Male, Intervention Group)

An interesting point about the decision to be a caregiver is that no participant expressed an awareness of caring for a limited acute period of time. Thus, it is unknown how long caregivers perceive the responsibility of caring for a person with hip fracture lasts for.

c) Transition from hospital to home

Caregivers expressed frustration, confusion, and uncertainty at the point of transitioning home. Such feelings were principally attributed to a lack of communication throughout discharge planning with caregivers feeling isolated, under-prepared and excluded from the decision-making. Visitor restrictions due to the COVID-19 pandemic, may have also reduced communication channels and

For peer review only - http://bmjopen.bmj.com/site/about/guidelines.xhtml

contributed to caregiver uncertainty, resulting in concerns of whether the person with hip fracture was receiving adequate.

"It's very difficult. I was at one point sending her back to hospital 'cause I was so frantic with it all." (Caregiver, Female, Intervention Group)

"Nothing is said [about discharge] at the hospital, apart from telling you have an appointment in eight weeks, there's nothing else said." (Caregiver, Male, Intervention Group)

"I mean the one thing that I do remember is really actually a feeling of isolation and a little bit concerned at whether or not I was doing the right things to support her." (Caregiver, Male, Intervention Group)

The profile of caregivers also contributes to their competence in managing the transition home, potentially relying on previous experiences to navigate the health system and available services.

"You know, imagine if you if you weren't an old nurse like me? How on earth can you even navigate the system? I think people are getting lost out there and it's creating a lot of problems for the health service." (Caregiver, Female, Intervention Group)

Some participants shared how they had to 'beg, steal and borrow' equipment from friends and family. It is apparent that the onus is on the caregiver themselves to navigate and seek the appropriate provisions for recovering at home with hip fracture and there was a feeling of abandonment.

"Luckily, some family had a spare single bed. A friend had a perching stool and a trolley. And then another friend, sadly her husband had been ill, but they had purchased their own wheelchair and we actually borrowed that wheelchair. And a cousin, he built some ramps so I could get the wheelchair in." (Caregiver, Female, Control Group)

DISCUSSION

Previous studies have provided important insights into the experiences of patients and caregivers following hip fracture, which we have further explored to better understand this challenge [13,28]. Our study aimed to understand the perspectives of informal caregivers who support the recovery of people following hip fracture surgery. Findings indicate that there is a tension between the expectations, and reality of the caregiving role for people with hip fracture. This is largely attributed to caregivers feeling disempowered to advocate for a person's recovery, leading to increased stress, anxiety and frustration when supporting them. Additionally, caregivers struggle with 'juggling' their own life with their caregiving role (e.g., full-time employment [29], whilst also maintaining their own health and well-being, for the benefit of both members of the dyad when discharged home [30]. There is a growing body of literature suggesting the mental health and wellness of a caregiver is also linked with a patient's functional and health outcomes [30–32]. Maintaining physical and mental ability to care for an individual is therefore important for hip fracture recovery.

This study identified that discharge processes after hip fracture do not currently fully prepare informal caregivers for their roles. As such, key learning points for health professionals are suggested to improve collaboration and support in the transition from hospital to home. These include: a greater explanation on 'normal' recovery processes; engaging for (dyad) joint decision-making; and promote the provision of additional (community) services for support. This is supported by previous findings

For peer review only - http://bmjopen.bmj.com/site/about/guidelines.xhtml

that have also highlighted several key recommendations for translational care which emphasise dyad engagement, education, and wellbeing [33,34].

Joint decision-making based on setting realistic and tailored goals, is another practical strategy health professionals may employ to support the transition of people with hip fracture and their informal caregiver from hospital to home. This agrees with previous authors such as Angeli et al [35] and Brewer et al [36] and re-enforced in Saletti-Cuesta et al's [13] systematic review, who stressed that collaborative goal setting can allow designated time to enhance family involvement, to focus on functional outcomes and highlight current priorities for the caregiving dyad. It therefore may be that informal caregiver are well-placed to act as a 'driver of goal-setting', as they may have intimate knowledge of the person pre-hip fracture (for example, preferences and character).

A key finding of this study was the powerlessness which caregivers felt they had in supporting and advocating for the person with hip fracture. As a result of their inability to advocate for the person with hip fracture, participants frequently reported feelings of either over anticipation (negative) contrasted with unrealistic (positive) opinions on what could be offered and provided to the person who they were supported. This is a common theme that resonates within previous literature [18,37,38]. Tutton et al [28] acknowledged the uncertainties shared by patients and caregivers after hip fractures and change of relationship within the caregiving dyad through disability. There was an expectation by health professionals did not sufficiently appreciate this uncertainty and particularly following a change in health status. Our findings suggest that health professionals expected that caregivers could navigate the health and social care system, which would indeed, require some level of health literacy. It is known that low health literacy among caregivers has the potential to impact adequate care provision [39]. This is particularly pertinent among those who are 'new' caregivers, those who may perceive to be particularly under-skilled, under-prepared and/or be adjusting to a new role or identify as an informal caregiver. This is contrary to experienced caregiver who could elicit competence, knowledge, and tolerance in caregiving [40–43]. Additionally, the profile of a caregiver may include a natural affinity to caring, namely a 'caring nature' [29,44]. There is therefore the need to develop interventions that improve informal caregiver health literacy, ultimately upskilling informal caregivers to have the confidence to advocating for the person with hip fracture.

This study offers insights into an informal caregiver population which may differ to most previously reported. Previous literature has emphasised the caregiver experience of caring for people with long-term conditions which is unlikely to be transferable to the acute, traumatic, and rapid nature of caregiving for people with hip fracture [45]. This study therefore offers unique perspectives on a different transition in becoming a (informal) caregiver, particularly as the findings suggest little time for decision-making and the assumption of accepting this role of significant responsibility [46]. A second element to this caregiving population is how it is gendered. Caregiving is known to have a greater adverse effect on female caregivers' mental health and life satisfaction [47]. However, the demographic of hip fracture is largely female (considering experiences of the menopause and osteoporosis, to name a few), as seen in our larger study necessitating male partners to take on the caring role who may find undertaking sensitive tasks more challenging [45,48]. Additionally, the qualities and skills frequently offered by female caregivers previously reported are that females are better communicators, better instigators of help and are better organised, suggesting that male caregivers might benefit with tailored support for these potential challenges [44]. Whilst it is recognised that expertise in managing frail, older patients is crucial to ensure holistic care for hip fracture patients [49], our study also featured much younger hip fracture participants, who had quite different perceptions of what was important to them and expectations of recovery [29,50].

This study presents with strengths and limitations. A notable strength was its ability to capture experiences during a novel time in healthcare history, where hospitals were making rapid, visitor policy changes in response to the COVID-19 pandemic. Due to the timing of the study, the participants

For peer review only - http://bmjopen.bmj.com/site/about/guidelines.xhtml

shared a distinctive experience and its effect on rehabilitation and on the caregiver experience. However, one may also interpret this as weakness as it is not a typical reflection of care for this population. Interviews took place within four-months post-hip fracture, despite previous literature suggesting physical recovery may take up to 12 months [51], and high levels of caregiver burden reported by caregivers at six months post-surgery [37]. There is currently little data regarding caregiver perspectives on the long-term recovery/care of hip fracture (for example, deconditioning and confidence in performing activities), thus understanding perspectives of the different caregiving trajectory, as people become more experienced carers and as people recover from hip fracture, would be valuable. Furthermore, it is unknown how long caregivers perceive the responsibility of caring for a person with hip fracture lasts for. No participant expressed an awareness of caring for an acute period of time and therefore how long this might last [52].

It is likely that group allocation (i.e., receiving the HIP HELPER intervention) may have impacted people's experiences. For example, the HIP HELPER workbook was designed to support goal-setting, thus such guidance may have influenced the way in-which recovering from, or caring for someone with a hip fracture may be perceived by participants. We acknowledge findings may not be fully generalisable because of the small sample size and England-based focus. Participants presented with limited socioeconomic, cultural or ethic perspectives, despite our best endeavours and there was also a lack of perspectives of those caring for a person with cognitive impairment [53]. This is important as such differences may be a source of variation in attitudes, behaviours, and perspectives of caregiving for someone following hip fracture [54,55]. We also appreciate that the present cohort was largely of lower mean age than may be expected for a hip fracture population, however this work provides more novel insights into the experiences of caregiving for younger people which is a profile anecdotally noted in practice [56]. Interviews were undertaken with informal caregivers and people with hip fracture together. This has limitations in that some individuals may be reluctant to address difficulties in the presence of the other person, some may assert dominance or reflect a particular status, and there is a likelihood of dyad interviews to be 'off-topic', compared to one-to-one interviews [57].

CONCLUSIONS

Whilst there may be an expectation by informal caregivers will provide support for people following hip fracture, our study illuminates challenges faced due to the acute and traumatic nature of hip fracture. We suggest that joint decision-making and goal setting with health professionals, patients and their informal carers will enable better preparedness for the transition from hospital to home. This includes a greater explanation and tailoring of the recovery pathway especially for younger hip fracture patients.

For peer review only

Contributors: SHa, AW, TS, SHo, KP, RK, PA, PL, MCo, SEL, MCr researched the topic and devised the study. TS, SHa, RK, PA, AC, SHo, AW, KP, PL, MCo, SEL, MCr provided the first draft of the manuscript. SHa lead the qualitative study design. AW lead the qualitative research conduct. TS, SHa, AW, RK, PA, AC, SHo, KP, PL, MCo, SEL, MCr contributed equally to manuscript preparation. TS acts a guarantor.

Collaborators: The HIP HELPER Study Collaborators: Penny Clifford (Norfolk, PPI Representative), Lis Freeman (Norfolk, PPI Representative), Rene Gray (Principal Investigator – James Paget University Hospital NHS Trust), Yan Cunningham (Principal Investigator – City Hospitals Sunderland NHS Foundation Trust), Sarah Langford (Principal Investigator - Northumbria Healthcare NHS Foundation Trust), Dr Mark Baxter (Principal Investigator - University Hospital Southampton NHS Foundation Trust), Jessica Pawson – (Principal Investigator - Barts Health NHS Trust), Melissa Taylor (James Paget University Hospital NHS Trust), Anna Mellows (James Paget University Hospital NHS Trust), Kate Lacey (James Paget University Hospital NHS Trust), Alex Herring (City Hospital Sunderland NHS Foundation Trust), Diane Williams (Northumbria Healthcare NHS Foundation Trust), Anna Cromie (Northumbria Healthcare NHS Foundation Trust), Gail Menton (Northumbria Healthcare NHS Foundation Trust), Warren Corbett (University Hospital Southampton NHS Foundation Trust), Helen Jowett (University Hospital Southampton NHS Foundation Trust), Vishwanath Joshi (Barts Health NHS Trust), Maninderpal Matharu (Barts Health NHS Trust), Maria Baggot (University Hospital Southampton NHS Foundation Trust) and David Barker (University Hospital Southampton NHS Foundation Trust).

Oversight Committee Membership: Associate Professor Susan Dutton (Chair; University of Oxford, UK), Professor Opinder Sahota (University of Nottingham, UK), Dr Katie Sheehan (Kings College London, UK).

Funding: This project is funded by the National Institute for Health and Care Research (NIHR) under its Research for Patient Benefit (RfPB) Programme (Grant Reference Number NIHR200731). MCo and SHo role in this study was supported by the National Institute for Health and Care Research Oxford Biomedical Research Centre. SEL role in this study was supported by the National Institute for Health and Care Research Exeter Biomedical Research Centre. The views expressed are those of the author(s) and not necessarily those of the NIHR or the Department of Health and Social Care.

Competing interests: None declared.

Disclaimer: MCo and SHo are supported by the National Institute for Health Research (NIHR) Oxford Biomedical Research Centre (BRC). SEL is supported by the National Institute for Health and Care Research Exeter Biomedical Research Centre. The views expressed are those of the author(s) and not necessarily those of the NHS, the NIHR or the Department of Health and Social Care.

Ethics approval and consent to participate: North East - Newcastle & North Tyneside 1 Research Ethics Committee (20/NE/0213) Date: 16 March 2021. Written informed consent was obtained from all participants.

Trial sponsor: University of East Anglia (Research Sponsor, Research and Innovation Services, The Registry, University of East Anglia, Norwich, NR4 7TJ; Email: researchsponsor@uea.ac.uk) The views expressed are those of the author(s) and not necessarily those of the sponsor.

Provenance and Peer Review: Not commissioned; externally peer reviewed.

Data availability statement: The data that support the findings of this study are available from the corresponding author (AW) upon reasonable request. This includes access to the full protocol, and anonymised participant-level dataset.

For peer review only - http://bmjopen.bmj.com/site/about/guidelines.xhtml

Patient consent for publication: Not required.

For peer review only

For peer review only - http://bmjopen.bmj.com/site/about/guidelines.xhtml

REFERENCES

1 Lee H, Cook JA, Lamb SE, *et al.* The findings of a surgical hip fracture trial were generalizable to the UK national hip fracture database. *J Clin Epidemiol* 2021;**131**. doi:10.1016/j.jclinepi.2020.11.016

2 Baker PN, Salar O, Ollivere BJ, *et al.* Evolution of the hip fracture population: Time to consider the future? A retrospective observational analysis. *BMJ Open* 2014;**4**. doi:10.1136/bmjopen-2013-004405

3 Lawler K, Taylor NF, Shields N. Involving family members in physiotherapy for older people transitioning from hospital to the community: A qualitative analysis. *Disabil Rehabil* 2015;**37**. doi:10.3109/09638288.2014.996673

4 Griffin XL, Parsons N, Achten J, *et al.* Recovery of health-related quality of life in a United Kingdom hip fracture population: The warwick hip trauma evaluation - A prospective cohort study. *Bone and Joint Journal* 2015;**97-B**. doi:10.1302/0301-620X.97B3.35738

5 Seitz DP, Adunuri N, Gill SS, *et al.* Prevalence of Dementia and Cognitive Impairment Among Older Adults With Hip Fractures. J Am Med Dir Assoc. 2011;**12**. doi:10.1016/j.jamda.2010.12.001

6 Em S, Bozkurt M, Caglayan M, *et al.* Psychological health of caregivers and association with functional status of stroke patients. *Top Stroke Rehabil* 2017;**24**. doi:10.1080/10749357.2017.1280901

7 Stajduhar KI, Funk L, Toye C, *et al.* Part 1: Home-based family caregiving at the end of life: A comprehensive review of published quantitative research (1998-2008). Palliat Med. 2010;**24**. doi:10.1177/0269216310371412

8 Erickson ME. Effects of motivation, roles, coping strategies, and adaptations in relationships and personality on caretaking of elderly parents by midlife couples. Dissertation Abstracts International Section A: Humanities and Social Sciences. 2002;**62**.

9 Albertini M, Kohli M, Vogel C. Intergenerational transfers of time and money in European families: Common patterns - Different regimes? *J Eur Soc Policy* 2007;**17**. doi:10.1177/0958928707081068

10 Gérain P, Zech E. Informal Caregiver burnout? Development of a theoretical framework to understand the impact of caregiving. *Front Psychol* 2019;**10**. doi:10.3389/fpsyg.2019.01748

11 Sutter-Leve R, Passint E, Ness D, *et al.* The caregiver experience after stroke in a covid-19 environment: A qualitative study in inpatient rehabilitation. *Journal of Neurologic Physical Therapy* 2021;**45**. doi:10.1097/NPT.0000000000000336

12 Feast A, Moniz-Cook E, Stoner C, *et al.* A systematic review of the relationship between behavioral and psychological symptoms (BPSD) and caregiver well-being. Int Psychogeriatr. 2016;**28**. doi:10.1017/S1041610216000922

13 Saletti-Cuesta L, Tutton E, Langstaff D, *et al.* Understanding informal carers' experiences of caring for older people with a hip fracture: a systematic review of qualitative studies. *Disabil Rehabil* 2018;**40**:740–50. doi:10.1080/09638288.2016.1262467

14 Hawkins RJ, Jowett A, Godfrey M, *et al.* Poststroke Trajectories: The Process of Recovery Over the Longer Term Following Stroke. *Glob Qual Nurs Res* 2017;**4**:233339361773020. doi:10.1177/2333393617730209

15 Sheehan KJ, Smith TO, Martin FC, *et al.* Conceptual Framework for an Episode of Rehabilitative Care After Surgical Repair of Hip Fracture. *Phys Ther* 2019;**99**:276–85. doi:10.1093/ptj/pzy145

16 Smith T, Clark L, Khoury R, *et al.* A feasibility study to assess the design of a multicentre randomized controlled trial of the clinical and cost-effectiveness of a caregiving intervention for people following hip fracture surgery. *Bone Jt Open* 2021;**2**. doi:10.1302/2633-1462.211.BJO-2021-0136

For peer review only - http://bmjopen.bmj.com/site/about/guidelines.xhtml

17 Tong A, Sainsbury P, Craig J. Consolidated criteria for reporting qualitative research (COREQ): A 32-item checklist for interviews and focus groups. *International Journal for Quality in Health Care* 2007;**19**. doi:10.1093/intqhc/mzm042
18 Ariza-Vega P, Ortiz-Piña M, Kristensen MT, *et al.* High perceived caregiver burden for relatives of patients following hip fracture surgery. *Disabil Rehabil* 2019;**41**. doi:10.1080/09638288.2017.1390612
19 Wimo A, Wetterholm AL, Mastey V, *et al.* Evaluation of the resource utilization and caregiver time in Anti-dementia drug trials—a quantitative battery. In: *The Health Economics of Dementia*. 1998.
20 Braun V, Clarke V. One size fits all? What counts as quality practice in (reflexive) thematic analysis? *Qual Res Psychol* 2021;**18**:328–52. doi:10.1080/14780887.2020.1769238
21 Charmaz C. *Constructing Grounded Theory*. 2nd ed. 2014.
22 Hanson S, Gilbert D, Landy R, *et al.* Cancer risk in socially marginalised women: An exploratory study. *Soc Sci Med* 2019;**220**:150–8. doi:10.1016/j.socscimed.2018.11.009
23 Skivington K, Matthews L, Simpson SA, *et al.* A new framework for developing and evaluating complex interventions: Update of Medical Research Council guidance. *The BMJ* 2021;**374**. doi:10.1136/bmj.n2061
24 Braun V, Clarke V. To saturate or not to saturate? Questioning data saturation as a useful concept for thematic analysis and sample-size rationales. *Qual Res Sport Exerc Health* 2021;**13**:201–16. doi:10.1080/2159676X.2019.1704846
25 Mauthner NS, Doucet A. Reflexive Accounts and Accounts of Reflexivity in Qualitative Data Analysis. *Sociology* 2003;**37**:413–31. doi:10.1177/00380385030373002
26 Hagens V, Dobrow MJ, Chafe R. Interviewee Transcript Review: assessing the impact on qualitative research. *BMC Med Res Methodol* 2009;**9**:47. doi:10.1186/1471-2288-9-47
27 Thomas DR. Feedback from research participants: are member checks useful in qualitative research? *Qual Res Psychol* 2017;**14**:23–41. doi:10.1080/14780887.2016.1219435
28 Tutton E, Saletti-Cuesta L, Langstaff D, *et al.* Patient and informal carer experience of hip fracture: a qualitative study using interviews and observation in acute orthopaedic trauma. *BMJ Open* 2021;**11**:e042040. doi:10.1136/bmjopen-2020-042040
29 Zarzycki M, Seddon D, Bei E, *et al.* Why do they care? A qualitative systematic review and meta-synthesis of personal and relational motivations for providing informal care. *Health Psychol Rev* 2022;:1–33. doi:10.1080/17437199.2022.2058581
30 Cobley CS, Fisher RJ, Chouliara N, *et al.* A qualitative study exploring patients' and carers' experiences of Early Supported Discharge services after stroke. *Clin Rehabil* 2013;**27**. doi:10.1177/0269215512474030
31 Isik AT, Soysal P, Solmi M, *et al.* Bidirectional relationship between caregiver burden and neuropsychiatric symptoms in patients with Alzheimer's disease: A narrative review. In: *International Journal of Geriatric Psychiatry*. 2019. doi:10.1002/gps.4965
32 Kuzuya M, Enoki H, Hasegawa J, *et al.* Impact of caregiver Burden on adverse health outcomes in community-dwelling dependent older care recipients. *American Journal of Geriatric Psychiatry* 2011;**19**. doi:10.1097/JGP.0b013e3181e9b98d
33 Naylor MD, Shaid EC, Carpenter D, *et al.* Components of Comprehensive and Effective Transitional Care. *J Am Geriatr Soc* 2017;**65**. doi:10.1111/jgs.14782
34 Guilcher SJT, Everall AC, Cadel L, *et al.* A qualitative study exploring the lived experiences of deconditioning in hospital in Ontario, Canada. *BMC Geriatr* 2021;**21**. doi:10.1186/s12877-021-02111-2
35 Angeli JM, Harpster KL, Hanson E, *et al.* Patient- and caregiver-identified preferences: Dimensions of change in developmental therapy treatment goals. *Dev Neurorehabil* 2019;**22**. doi:10.1080/17518423.2018.1425754

For peer review only - http://bmjopen.bmj.com/site/about/guidelines.xhtml

36 Brewer K, Pollock N, Wright FV. Addressing the challenges of collaborative goal setting with children and their families. *Phys Occup Ther Pediatr* 2014;**34**. doi:10.3109/01942638.2013.794187

37 Parry JA, Langford JR, Koval KJ. Caregivers of hip fracture patients: The forgotten victims? *Injury* 2019;**50**. doi:10.1016/j.injury.2019.09.030

38 Ding TYG, De Roza JG, Chan CY, *et al.* Factors associated with family caregiver burden among frail older persons with multimorbidity. *BMC Geriatr* 2022;**22**. doi:10.1186/s12877-022-02858-2

39 Yuen EYN, Knight T, Ricciardelli LA, *et al.* Health literacy of caregivers of adult care recipients: A systematic scoping review. Health Soc Care Community. 2018;**26**. doi:10.1111/hsc.12368

40 Kim Y. Korean-American family postcaregivers on dementia caregiving: A phenomenological inquiry. *J Gerontol Soc Work* 2009;**52**. doi:10.1080/01634370903048352

41 Kuşçu MK, Dural U, Yaşa Y, *et al.* Decision pathways and individual motives in informal caregiving during cancer treatment in Turkey. *Eur J Cancer Care (Engl)* 2009;**18**. doi:10.1111/j.1365-2354.2007.00900.x

42 Øydgard GW. The influence of institutional discourses on the work of informal carers: An institutional ethnography from the perspective of informal carers. *BMC Health Serv Res* 2017;**17**. doi:10.1186/s12913-017-2591-7

43 Stajduhar KI, Martin WL, Barwich D, *et al.* Factors influencing family caregivers' ability to cope with providing end-of-life cancer care at home. *Cancer Nurs* 2008;**31**. doi:10.1097/01.NCC.0000305686.36637.b5

44 Zarzycki M, Morrison V, Bei E, *et al.* Cultural and societal motivations for being informal caregivers: a qualitative systematic review and meta-synthesis. *Health Psychol Rev* Published Online First: 2022. doi:10.1080/17437199.2022.2032259

45 Bueno M, Chase J-A. Gender Differences in Caregiving of Older Adults: A Systematic Review of the Literature. *Innov Aging* 2020;**4**. doi:10.1093/geroni/igaa057.491

46 Boonsin S, Deenan A, Wacharasin C. Factors influencing the burden of family caregiving for survivors of stroke. *Pac Rim Int J Nurs Res Thail* 2020;**25**.

47 Le DD, Ibuka Y. Understanding the effects of informal caregiving on health and well-being: Heterogeneity and mechanisms. *Soc Sci Med* 2023;**317**:115630. doi:10.1016/j.socscimed.2022.115630

48 Alpantaki K, Papadaki C, Raptis K, *et al.* Gender and Age Differences in Hip Fracture Types among Elderly: a Retrospective Cohort Study. *Maedica (Bucur)* 2020;**15**. doi:10.26574/maedica.2020.15.2.185

49 Fox F, Drew S, Gregson C, *et al.* Complex organisational factors influence multidisciplinary care for patients with hip fractures: a qualitative study of barriers and facilitators to service delivery. *BMC Musculoskelet Disord* 2023;**24**:128. doi:10.1186/s12891-023-06164-9

50 Griffiths F, Mason V, Boardman F, *et al.* Evaluating recovery following hip fracture: a qualitative interview study of what is important to patients. *BMJ Open* 2015;**5**:e005406–e005406. doi:10.1136/bmjopen-2014-005406

51 Magaziner J, Chiles N, Orwig D. Recovery after Hip Fracture: Interventions and Their Timing to Address Deficits and Desired Outcomes-Evidence from the Baltimore Hip Studies. *Nestle Nutr Inst Workshop Ser* 2015;**83**. doi:10.1159/000382064

52 Cameron JI, Gignac MAM. "Timing It Right": A conceptual framework for addressing the support needs of family caregivers to stroke survivors from the hospital to the home. *Patient Educ Couns* 2008;**70**:305–14. doi:10.1016/j.pec.2007.10.020

53 Francis N, Hanna P. Informal carer experiences of UK dementia services—A systematic review. J Psychiatr Ment Health Nurs. 2022;**29**. doi:10.1111/jpm.12698

54 Fernández-Ballesteros R, Sánchez-Izquierdo M, Olmos R, *et al.* Cultural stereotypes in care contexts. *Clin Interv Aging* 2018;**13**. doi:10.2147/CIA.S169487

For peer review only

For peer review only - http://bmjopen.bmj.com/site/about/guidelines.xhtml

55 Dilworth-Anderson P, Williams IC, Gibson BE. Issues of race, ethnicity, and culture in caregiving research: A 20-year review (1980-2000). Gerontologist. 2002;**42**. doi:10.1093/geront/42.2.237
56 Veronese N, Maggi S. Epidemiology and social costs of hip fracture. *Injury* 2018;**49**. doi:10.1016/j.injury.2018.04.015
57 Szulc J, King N. The Practice of Dyadic Interviewing: Strengths, Limitations and Key Decisions. *Forum Qualitative Sozialforschung Forum: Qualitative Social Research* 2022;**23**.

For peer review only

Table 1. Characteristics of sample interviewed

	Intervention group N=7	Control group N=3	Overall N=10
**Informal caregiver**			
Age (years): median (IQR)	71.0 (58.0, 81.0)	71.0 (43.0, 72.0)	71.0 (58.0, 77.0)
Gender: n (%)			
- Male	5 (71.4%)	2 (66.7%)	7 (70.0%)
- Female	2 (28.6%)	1 (33.3%)	3 (30.0%)
Ethnicity: n (%)			
- White British	6 (85.7%)	3 (100%)	9 (90.0%)
- White other	1 (14.3%)	0	1 (10.0%)
Relationship to person with hip fracture: n (%)	4 (57.1%)	2 (66.7%)	6 (60.0%)
- Spouse	1 (14.3%)	1 (33.3%)	2 (20.0%)
- Daughter/son	2 (28.6%)	0	2 (20.0%)
- Other			
Employment: n (%)			
- Not working	6 (85.7%)	3 (100%)	9 (90.0%)
- Part-time work	1 (14.3%)	0	1 (10.0%)
**Person with hip fracture**			
Age at consent (years): median (IQR)	79.0 (70.0, 82.0)	69.0 (68.0, 71.0)	72.5 (69.0, 79.0)
Gender: n (%)			
- Male	1 (14.3%)	0	1 (10.0%)
- Female	6 (85.7%)	3 (100%)	9 (90.0%)
Ethnicity: n (%)			
- White British	7 (100%)	3 (100%)	10 (100%)
Has Cognitive Impairment (based on AMTS category): n (%)	1 (14.3%)	0	1 (10.0%)
AMTS score at consent: median (IQR)	10.0 (9.0, 10.0)	10.0 (10.0, 10.0)	10.0 (9.0, 10.0)
NEADL score at baseline: median (IQR)	20.0 (14.0, 22.0)	10.0 (10.0, 10.0)	17.0 (12.0, 22.0)

**Location**		
Site (n)		
1	1	1
2	1	1
3	1	1
4	3	-
5	1	-

AMTS – Abbreviated Mental Test Score; NEADL – Nottingham Extended Activities of Daily Living scale

For peer review only

Topic	Sample questions	Prompts/probes
Overall	• Overall, do share your experiences of being involved with our research?	
The approach and consent process	• Please talk me through how you got involved with the HipHelper research study?<br>• How clearly was the study explained to you?	• Could we have dealt with that differently?<br>• How was this different to what you expected?<br>• For the future, what could we improve about the description of the study?
Willingness to be randomised to either group/ participant views of their intervention<br><br>The risk of intervention contamination between the groups	• Please talk me through what study treatment you received<br><br>• How much did talk to any other patients or caregivers (whilst in hospital) about the intervention?	• *If required: clarify what HIP HELPER was and what was usual care/non-study intervention*<br>• You were allocated to X group. What did that feel like?<br>• What discussions were had between those who received it and did not receive it?
**Both groups**		
The acceptability of the inpatient care	• Please talk me through your treatment while you were in the hospital	• What aspects of your care that were lacking in the hospital?<br>• Can you remember which wards you were on? Can you remember being moved to different areas of the hospital?

For peer review only

Topic	Sample questions	Prompts/probes
	• *(Carers)* As X's carer, what was your impression of the care?	• For both of you, what was helpful and less helpful to your care?
**Intervention group only**		
In-patient HIP HELPER programme<br><br>Strengths/weaknesses	• How did you find the HIP HELPER programme?<br><br>• Please tell me what you remember about the manual handling training you had in the hospital?<br>• Please tell me about the phone calls you received?<br><br>• What were the individualised goals you set in the hospital?	• Please share any specific examples of what you can remember from the programme?<br>• What were the most helpful bits of your HIP HELPER intervention?<br>• What was good about it?<br>• What were the less helpful/worse bits of the HIP HELPER intervention?<br>• For you as the patient/and you as X's carer?<br>• How did you get on with that at home?<br>• Was it helpful? If so, how?<br><br>• Can you remember what you talked about?<br>• Can you give specific examples of what was helpful?<br>• Was there any advice that confused you or you weren't clear about?<br>• How did you come up with these?<br>• *(Carer)* By how far were you involved in the goal setting process?<br>• In retrospect, what goal(s) would you alter?

For peer review only

**Topic**	**Sample questions**	**Prompts/probes**
**Both groups:**		
On return to your home	• *(If required, state residential status)* Can you tell me who you live with at home?	
	• What were the first things you wanted to achieve once at home?<br>• How did you decide who was to be your allocated carer?<br>• *(Carers)* Can you talk me through this decision to be designated as X's carer?	• On a 1-10 scale, how confident did you feel when you left the hospital?<br>• Can you tell me why you have chosen that number on the scale?
	• *(Long-term carers)* Once at home, how do you feel care-giving role changed?	• Can you tell me about any new caring responsibilities?<br>• How did you organise these?<br>• Did this change the dynamics in their relationship in taking on caring role, if so, how
	• What sort of adaptions did you make at home to enable you to move about independently (where possible)?<br>• What do you think is lacking in the transition from hospital to home?	• Are/were there any other things that may have affected your transfer home?<br>• Are/were there any other things that required you to adapt the advice you were given?
	• How willing were/are you to continue with activities/advice that were suggested to you?<br>• Once at home, by how far did you feel supported by the health care professions you had seen?	*Probes: transferring, dressing, activities of daily living*

For peer review only

Topic	Sample questions	Prompts/probes
	• *(Carers)* For you as caregiver, how did you get on with helping move X about?<br>• Were there any adaptions that you made to the advice given?	
What modifications they may recommend to interventions received	• What changes did you find easy to implement and what did you find harder?	• What could we improve?
	• What do we need to modify for any future programmes to support people with a hip replacement and their carer to help you get up and moving and returning to normal life?	• Can you explain why? *Prompt around ADL's*
The ease and convenience of the data collection processes/applicability of the methods and measures used	• As you were part of a trial, we had to collect a lot of measurements. Can you talk me through what these were?	*Prompt:*<br>*'Share screen' of the front page of questionnaires to help with memory recall.*
	• How did you manage with the questionnaires we gave you at the start of the study and at the end in the post?<br>• What about your interview experience today?	• Were they easy to complete?<br>• Do you remember them being a problem?<br>• How convenient were they?<br>• How did you find it?<br>• Is there anything we can do to improve this experience?
Summary and end	• We are now ? months since your hip replacement, by how far do you think the HipHelper programme (*or usual care)* has helped with your recovery?<br>• If we were to run this programme again in the future, is there anything else we should consider?	• How far do you think the Hip Helper programme (*or usual care)* has helped you achieve your goals?

For peer review only

Topic	Sample questions	Prompts/probes
	• How do you think we could better support you and your carer to recover after hip surgery?<br>• Is anything else you have gained by being involved in this research study<br>• Is there anything else we haven't thought of and that you can share with us to improve the study and the care of hip replacement patients and their carers?	

For peer review only

COREQ (COnsolidated criteria for REporting Qualitative research) Checklist

A checklist of items that should be included in reports of qualitative research. You must report the page number in your manuscript where you consider each of the items listed in this checklist. If you have not included this information, either revise your manuscript accordingly before submitting or note N/A.

Topic	Item No.	Guide Questions/Description	Reported on Page No.
**Domain 1: Research team and reflexivity**			
Personal characteristics			
Interviewer/facilitator	1	Which author/s conducted the interview or focus group?	
Credentials	2	What were the researcher's credentials? E.g. PhD, MD	
Occupation	3	What was their occupation at the time of the study?	
Gender	4	Was the researcher male or female?	
Experience and training	5	What experience or training did the researcher have?	
Relationship with participants			
Relationship established	6	Was a relationship established prior to study commencement?	
Participant knowledge of the interviewer	7	What did the participants know about the researcher? e.g. personal goals, reasons for doing the research	
Interviewer characteristics	8	What characteristics were reported about the inter viewer/facilitator? e.g. Bias, assumptions, reasons and interests in the research topic	
**Domain 2: Study design**			
Theoretical framework			
Methodological orientation and Theory	9	What methodological orientation was stated to underpin the study? e.g. grounded theory, discourse analysis, ethnography, phenomenology, content analysis	
Participant selection			
Sampling	10	How were participants selected? e.g. purposive, convenience, consecutive, snowball	
Method of approach	11	How were participants approached? e.g. face-to-face, telephone, mail, email	
Sample size	12	How many participants were in the study?	
Non-participation	13	How many people refused to participate or dropped out? Reasons?	
Setting			
Setting of data collection	14	Where was the data collected? e.g. home, clinic, workplace	
Presence of non-participants	15	Was anyone else present besides the participants and researchers?	
Description of sample	16	What are the important characteristics of the sample? e.g. demographic data, date	
Data collection			
Interview guide	17	Were questions, prompts, guides provided by the authors? Was it pilot tested?	
Repeat interviews	18	Were repeat inter views carried out? If yes, how many?	
Audio/visual recording	19	Did the research use audio or visual recording to collect the data?	
Field notes	20	Were field notes made during and/or after the inter view or focus group?	
Duration	21	What was the duration of the inter views or focus group?	
Data saturation	22	Was data saturation discussed?	
Transcripts returned	23	Were transcripts returned to participants for comment and/or	

For peer review only

Topic	Item No.	Guide Questions/Description	Reported on Page No.
		correction?	
**Domain 3: analysis and findings**			
Data analysis			
Number of data coders	24	How many data coders coded the data?	
Description of the coding tree	25	Did authors provide a description of the coding tree?	
Derivation of themes	26	Were themes identified in advance or derived from the data?	
Software	27	What software, if applicable, was used to manage the data?	
Participant checking	28	Did participants provide feedback on the findings?	
Reporting			
Quotations presented	29	Were participant quotations presented to illustrate the themes/findings? Was each quotation identified? e.g. participant number	
Data and findings consistent	30	Was there consistency between the data presented and the findings?	
Clarity of major themes	31	Were major themes clearly presented in the findings?	
Clarity of minor themes	32	Is there a description of diverse cases or discussion of minor themes?	

Developed from: Tong A, Sainsbury P, Craig J. Consolidated criteria for reporting qualitative research (COREQ): a 32-item checklist for interviews and focus groups. *International Journal for Quality in Health Care*. 2007. Volume 19, Number 6: pp. 349 – 357

Once you have completed this checklist, please save a copy and upload it as part of your submission. DO NOT include this checklist as part of the main manuscript document. It must be uploaded as a separate file.

For peer review only
